# Estrogen-mediated downregulation of HIF-1α signaling in B lymphocytes influences postmenopausal bone loss

**DOI:** 10.1038/s41413-022-00189-x

**Published:** 2022-02-17

**Authors:** Xianyi Meng, Zhen Lin, Shan Cao, Iga Janowska, Koshiro Sonomoto, Darja Andreev, Knab Katharina, Jinming Wen, Karl Xaver Knaup, Michael Sean Wiesener, Gerhard Krönke, Marta Rizzi, Georg Schett, Aline Bozec

**Affiliations:** 1 Department of Internal Medicine 3, Friedrich-Alexander-University Erlangen-Nürnberg (FAU) and Universitätsklinikum Erlangen, Erlangen, 91054 Germany; 2 Deutsches Zentrum fur Immuntherapie (DYI), Erlangen, 91054 Germany; 3 Department of Rheumatology and Clinical Immunology, Faculty of Medicine, Medical Center-University of Freiburg, Freiburg, 79106 Germany; 4 Department of Internal Medicine 4, Friedrich-Alexander-University Erlangen-Nürnberg (FAU) and Universitätsklinikum Erlangen, Erlangen, 91054 Germany

**Keywords:** Bone, Osteoporosis

## Abstract

In the bone marrow, B cells and bone-resorbing osteoclasts colocalize and form a specific microenvironment. How B cells functionally influence osteoclasts and bone architecture is poorly understood. Using genetically modified mice and high-throughput analyses, we demonstrate that prolonged HIF-1α signaling in B cells leads to enhanced RANKL production and osteoclast formation. In addition, deletion of HIF-1α in B cells prevents estrogen deficiency-induced bone loss in mice. Mechanistically, estrogen controls HIF-1α protein stabilization through HSP70-mediated degradation in bone marrow B cells. The stabilization of HIF-1α protein in HSP70-deficient bone marrow B cells promotes RANKL production and osteoclastogenesis. Induction of HSP70 expression by geranylgeranylacetone (GGA) administration alleviates ovariectomy-induced osteoporosis. Moreover, *RANKL* gene expression has a positive correlation with *HIF1A* expression in human B cells. In conclusion, HIF-1α signaling in B cells is crucial for the control of osteoclastogenesis, and the HSP70/HIF-1α axis may serve as a new therapeutic target for osteoporosis.

## Introduction

The balance between bone formation by osteoblasts and bone resorption by osteoclasts regulates bone homeostasis.^1,2^ Osteoclast activation is a hallmark of many forms of bone loss. These multinucleated cells are differentiated from the monocyte/macrophage lineage, and their differentiation essentially depends on macrophage colony-stimulating factor (M-CSF) and receptor activator of nuclear factor kappa-Β ligand (RANKL).^3,4^ M-CSF allows the survival of monocytes, whereas RANKL promotes their differentiation into mature osteoclasts, which is further enhanced by many proinflammatory cytokines, including interleukin (IL)-6, IL-17, IL-1β, and TNF-α.^5–8^ To date, several cell types, such as T lymphocytes, B lymphocytes, chondrocytes, osteoblasts, and fibroblasts, have been shown to support osteoclast activation under physiological and pathological conditions through microenvironmental factors in bone marrow.^9–14^ However, the regulatory pathways for the production of these microenvironmental factors during osteoclastogenesis are not well understood.

B lymphocytes represent 30%–40% of bone marrow cells and share a common microenvironment with osteoclasts.^15^ Therefore, it is likely that B cell/osteoclast crosstalk contributes to bone homeostasis. IL-7 transgenic mice not only showed increased bone marrow B cells but also enhanced bone loss, while IL-7 receptor-deficient mice exhibited elevated bone mass together with impaired bone marrow B cell development.^16^ B cells can be an important source of RANKL as well as its decoy receptor OPG.^17–19^ For instance, mice with B cell-specific deletion of RANKL were partially protected from ovariectomy (OVX)-induced osteoporosis.^20^ Moreover, mice lacking tuberous sclerosis complex 1 in B cells are osteoporotic with a high level of RANKL but a low level of osteoprotegerin (OPG) expression.^21^ However, the intrinsic mechanism that functionally controls RANKL expression in B cells is still elusive.

B cells reside in the so-called “osteoblastic niches” in the bone marrow and are characterized by low oxygen concentrations.^22^ Hence, the development and proliferation of B cells in the bone niche occurs under low oxygen pressure requiring metabolic adaptation to the hypoxic microenvironment, which is controlled by the transcription factor hypoxia-inducible factor (HIF)-1α.^23^ During normoxia, HIF-1α is continuously hydroxylated by prolyl hydroxylase domain-containing enzymes (PHDs), which in turn recruits the von Hippel-Lindau protein (pVHL) and directs HIF-1α toward ubiquitination, mediating its proteasomal degradation.^24,25^ Under hypoxic conditions, PHD activities are inhibited, resulting in HIF-1α stabilization and dimerization with constitutively expressed HIF-1β. The heterodimerized HIF-1 complex binds to the hypoxia-responsive element (HRE) region and activates target gene expression.^26^ Although HIF-1α is regulated by PHDs and pVHL in an oxygen-dependent manner, recent studies have shown that heat shock proteins (HSPs) play an essential role in the regulation of HIF-1α protein levels in an oxygen-independent manner.^27,28^ Among HSPs that mediate protein folding, protein degradation, and protein function, HSP70 (*Hsp1a1*) and HSP90 (*Hsp90aa/b1*) are involved in proteasomal degradation through the ubiquitin–proteasome system and thus also regulate HIF-1α levels.^29–31^

In this study, we reported that sustained HIF-1α activation in bone marrow B cells triggers a pro-osteoclastogenic environment. Conversely, a lack of *Hif1a* in B cells restricts estrogen deficiency-induced bone loss. Molecularly, HIF-1α expression levels in B cells are controlled by estrogen-induced HSP70 expression through the ubiquitin–proteasome degradation system. As a result, genetic deletion of HSP70 in B cells markedly exacerbated HIF-1α signaling activation and RANKL-mediated osteoclastogenesis. Therefore, our findings suggest that the HSP70/HIF-1α axis may offer a novel therapeutic strategy for osteoporosis.

## Results

### Activation of HIF-1α signaling in B cells enhances osteoclastogenesis and induces osteoporosis

**Fig. 1:**
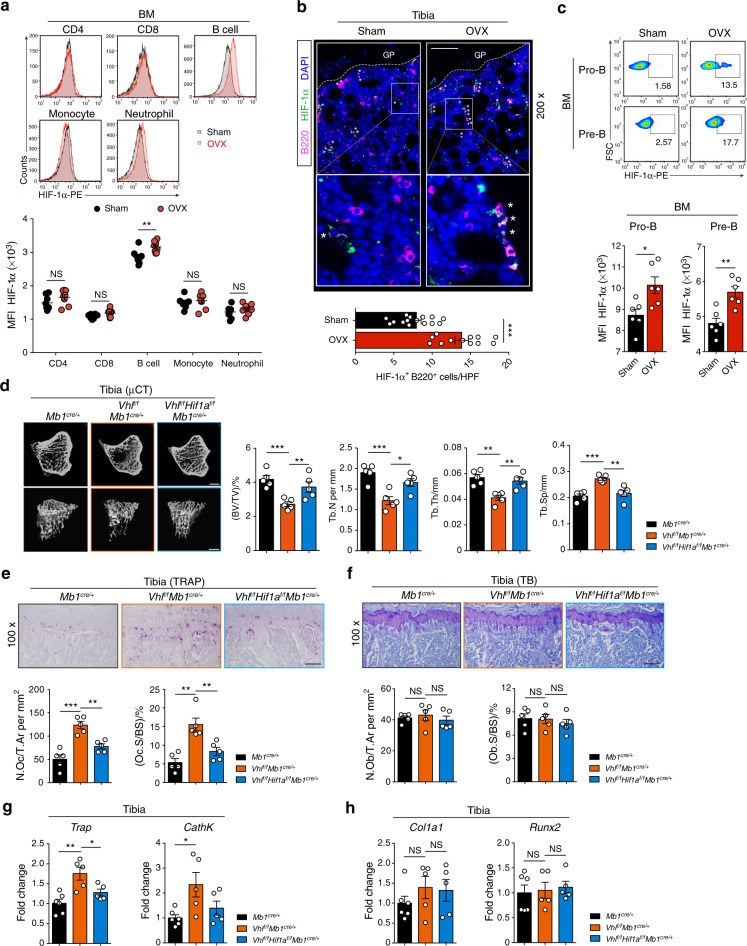
Sustained activation of HIF-1α signaling in B cells enhances osteoclastogenesis and osteoporosis. **a** Quantification and histograms of HIF-1α expression in CD4 T cells, CD8 T cells, B cells, monocytes and neutrophils from the bone marrow of sham-operated (Sham) and ovariectomized (OVX) mice (*n* = 8). **b** Representative immunofluorescence microscopy images of tibial sections from Sham and OVX mice (HIF-1α, green; B220, purple), along with the average number of HIF-1α^+^B220^+^ B cells per high-power field (HPF). Asterisks indicate HIF-1α^+^B220^+^ B cells in the bone niche. GP growth plate. Scale bars, 50 μm. **c** Representative plots and quantification of HIF-1α expression in Pro-B and Pre-B populations from Sham and OVX mice (*n* = 6). **d** Representative μCT images of tibial trabecular bone and structural parameters (BV/TV, Tb.N, Tb.Th, Tb.Sp) in *Mb1*^cre/+^, *Vhl*^f/f^*Mb1*^cre/+^ and *Vhl*^f/f^*Hif1a*^f/f^*Mb1*^cre/+^ mice (*n* = 5). Scale bars, 500 μm. Representative TRAP staining (**e**) and TB staining (**f**) in tibias from mice shown in (**d**). Bone resorption parameters (N.Oc/T.Ar, Oc.S/BS) and bone formation parameters (N.Ob/T.Ar, Ob.S/BS) in metaphyseal regions of the tibia were assessed by histomorphometric analyses. Scale bars, 100 μm. *Trap*, *CathK* (**g**), *Col1a1* and *Runx2* (**h**) expression in bone from mice shown in (**d**). Values for control group were set as 1. *P < 0.05, **P < 0.01, ***P < 0.001

Bone marrow, where B cells develop and osteoclasts reside, has low oxygen tension.^22^ The role of HIF-1α in controlling B cell/osteoclast interactions and bone homeostasis, however, is poorly defined. To investigate whether HIF-1α signaling is affected in bone marrow immune cells during estrogen deficiency-induced bone loss, HIF-1α expression in common lymphoid progenitors (CLPs), CD4 T cells, CD8 T cells, B cells, monocytes, and neutrophils from the bone marrow of ovariectomized and sham-operated wild-type (WT) mice was compared. Surprisingly, HIF-1α expression was upregulated only in bone marrow B cells after OVX (Fig. 1a, b) (Fig. S1a, b). Consistently, HIF-1α target gene expression was increased in B cells from the bone marrow of ovariectomized mice relative to sham controls (Fig. S1c). When separately analyzing B cell subpopulations in bone marrow and periphery, OVX surgery particularly induced HIF-1α expression in Pro-B cells (c-kit^+^CD43^+^CD19^+^B220^+^) and Pre-B cells (CD25^+^CD43^−^CD19^+^IgM^−^) (Fig. 1c) (Fig. S1d, e). Bioinformatic analysis of the transcriptome of bone marrow cells in the Immunological Genome Project revealed high estrogen intracellular signaling in Pro-B and Pre-B populations (Fig. S1f), including the estrogen receptor α gene (*Esr1*).^32^ In addition, high estrogen receptor α (ERα) protein expression levels were found in bone marrow B cells, especially in the Pro-B and Pre-B subpopulations (Fig. S1g, h), suggesting that these two populations are the major immune cell populations influenced by estrogen levels.

To further confirm whether HIF-1α activation in B cells affects bone homeostasis, we used *Mb1*-cre (*Mb1*^cre/+^) mice to generate B cell-specific conditional knockout mice. First, we characterized the specificity of the *Mb1*-cre line by breeding *Mb1*^cre/+^ mice to Ai14 tdTomato reporter mice. As expected, only the B cell population showed positivity for tdTomato expression. (Fig. S2a, b). Next, mice with B cell-specific deletion of *Vhl* (*Vhl*^f/f^*Mb1*^cre/+^) were generated. As expected, sustained HIF-1α signaling in B cells was observed in *Vhl* conditional knockout mice (Fig. S2c). Since HIF-1α is not the only substrate for the VHL protein, *Vhl*^f/f^*Hif1a*^f/f^*Mb1*^cre/+^ double knockout mice were also generated to address the specific role of HIF-1α signaling activation in bone homeostasis. Tibial bone parameters were evaluated in *Vhl*^fl/fl^*Mb1*^cre/+^ and control *Mb1*^cre/+^ mice by micro-computed tomography (μCT) analyses. *Vhl* deletion in B cells caused an osteopenic phenotype in 8-week-old female *Vhl*^f/f^*Mb1*^cre/+^ mice relative to WT controls (Fig. 1d). Bone loss in *Vhl*^f/f^*Mb1*^cre/+^ mice was coupled with increased TRAP^+^ osteoclast and osteoclast-specific gene expression, *Trap* and *CathK*, whereas no difference was found in osteoblast cells and the osteoblast-specific genes *Col1a1* and *Runx2* (Fig. 1e–h). Interestingly, B cell-specific deletion of *Hif1a* in the *Vhl*-deficient background reversed the osteopenic phenotype of the *Vhl*^f/f^*Mb1*^cre/+^ mice (Fig. 1d), which was consistent with the rescue of TRAP^+^ osteoclast number and osteoclast-specific gene expression in the double-deficient mice. Again, no significant difference was found in osteoblast parameters or osteoblast-specific gene expression (Fig. 1e–h). All these data suggest that activation of HIF-1α signaling in B cells enhances osteoclastogenesis and accelerates osteoporosis.

### HIF-1α directly regulates *Rankl* gene expression

**Fig. 2:**
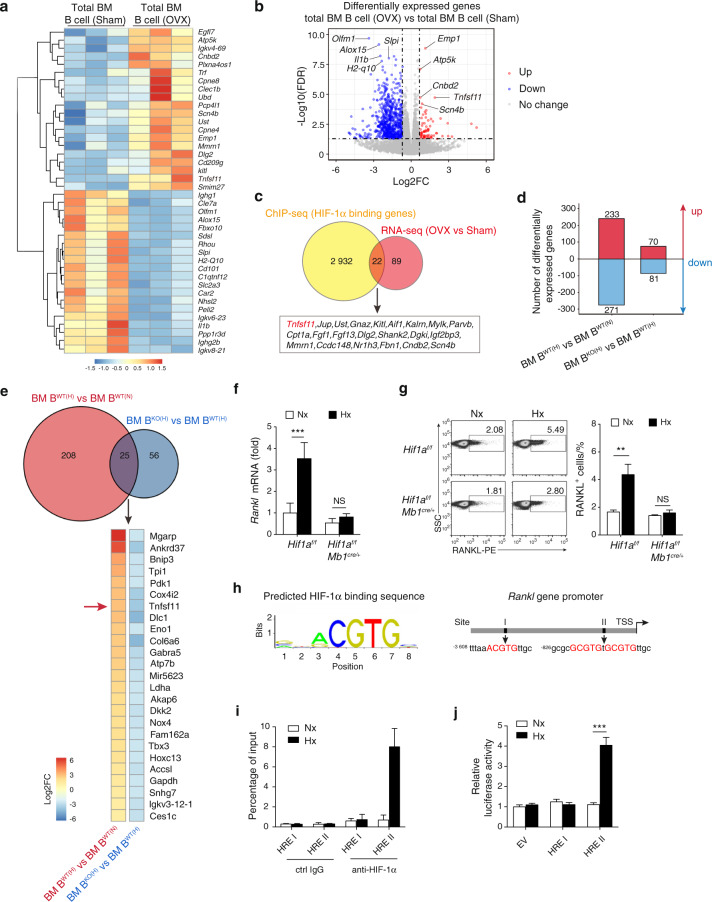
HIF-1α signaling activation in B cells enhances RANKL production. **a** Differentially expressed genes in total B cells from bone marrow of sham control (Sham) or ovariectomized (OVX) mice (*n* = 3). **b** Volcano plot of RNA-seq analysis of total B cells from bone marrow of Sham and OVX mice showing upregulated and downregulated genes (*n* = 3). **c** Experimental outline that led to the discovery of RANKL (*Tnfsf11*) as a HIF-1α target gene during ovariectomy-induced bone loss. **d** Number of differentially expressed genes in WT and *Hif1a*-deficient bone marrow B cells after normoxic or hypoxic culture for 12 h. WT B cells under hypoxia, B^WT (H)^; WT B cells under normoxia, B^WT (N)^; *Hif1a*-deficient B cells under hypoxia, B^KO (H)^; *Hif1a*-deficient B cells under normoxia, B^KO (N)^. **e** Venn diagram depicting overlap of differentially expressed genes between hypoxic WT B cells versus normoxic WT B cells (red circle) and hypoxic *Hif1a*-deficient B cells versus hypoxic WT B cells (blue circle). Heatmap showing the commonly differentially expressed genes. Color scale indicates Log_2_ fold change. **f** *Rankl* mRNA expression in *Hif1a*-deficient B cells or control B cells after normoxic (Nx) or hypoxic (Hx) culture for 12 h (*n* = 6). **g** RANKL expression in B cells isolated from the bone marrow of *Hif1a*^f/f^ or *Hif1a*^f/f^*Mb1*^cre/+^ mice after normoxic (Nx) or hypoxic (Hx) culture for 24 h (*n* = 6). **h** Transcription factor HIF-1α binding sequence in the JASPAR database (left) and schematic analysis of hypoxia response elements (HREs) on the *Rankl* promoter (right). **i** ChIP assays showing the binding of HIF-1α to the *Rankl* promoter in total bone marrow B cells after normoxic (Nx) or hypoxic (Hx) culture for 12 h (*n* = 3). **j** Luciferase reporter assay in CH12F3 cells transfected with empty vector (EV) or HRE constructs (I and II) after normoxic (Nx) or hypoxic (Hx) culture for 24 h (*n* = 3). **P < 0.01, ***P < 0.001

To understand how HIF-1α signaling modulates B cells during estrogen deficiency-induced bone loss, RNA sequencing (RNA-seq) analysis and HIF-1α ChIP-sequencing (ChIP-seq) analysis were performed on bone marrow B cells from mice that received sham or OVX surgery. Out of the 111 upregulated genes and the 2954 HIF-1α binding genes under estrogen-deficient conditions, we identified 22 genes (*Tnfsf11*, *Jup*, *Ust*, *Gnaz*, *Kitl*, *Aif1*, *Kalm*, *Mylk*, *Parvb*, *Cpt1a*, *Fgf1*, *Fgf13*, *Dlg2*, *Shank2*, *Dgki*, *Igf2bp3*, *Mmrn1*, *Ccdc148*, *Nr1h3*, *Fbn1*, *Cndb2*, *Scn4b*), including *Rankl (Tnfsf11)*, that were transcriptionally regulated by HIF-1α in total bone marrow B cells during estrogen deficiency (Fig. 2a–c) (Table S1). To further dissect whether *Rankl* expression in bone marrow B cells was dependent on HIF-1α expression, WT and *Hif1a*-deficient bone marrow B cells were cultured under normoxic or hypoxic conditions. RNA-seq analysis identified vast number of genes upregulated in WT B cells under hypoxic culture relative to WT B cells under normoxic culture, whereas a much smaller number of genes reduced in *Hif1a*-deficient B cells relative to control B cells with hypoxic culture (Fig. 2d). *Rankl (Tnfsf11)* was one of the genes increased in WT B cells under hypoxia but to a much lesser extent in *Hif1a*-deficient B cells (Fig. 2e). Quantitative RT–PCR and flow cytometry confirmed that hypoxia-induced HIF-1α signaling led to increased RANKL mRNA and protein levels (Fig. 2f, g). Moreover, increased RANKL expression was abolished in *Hif1a*-deficient bone marrow B cells under hypoxic conditions, which indicates that hypoxia-induced RANKL production by B cells is dependent on HIF-1α signaling (Fig. 2f, g).

Since HIF-1α is a potent transcription factor, we investigated whether *Rankl* gene expression is transcriptionally regulated by HIF-1α. Bioinformatics promoter analysis using the JASPAR database with the HRE consensus core (A/GCGTG) revealed two HRE putative regions (I, II) in the *Rankl* promoter (Fig. 2h). We showed that HIF-1α bound to the HRE II region under hypoxic conditions using a chromatin immunoprecipitation (ChIP) assay (Fig. 2i). Next, luciferase reporter assays with putative HRE constructs were performed in the B cell line CH12F3. As expected, only the luciferase activity of the HRE II construct was increased under hypoxic conditions, suggesting that HIF-1α induces *Rankl* transcription by binding to the HRE II region (Fig. 2j). Collectively, these data demonstrate that HIF-1α directly binds to the *Rankl* promoter and regulates RANKL expression in bone marrow B cells during estrogen deficiency-induced osteoporosis.

### Deletion of HIF-1α in B cells partially protects against ovariectomy-induced osteoporosis through RANKL-mediated osteoclastogenesis

**Fig. 3:**
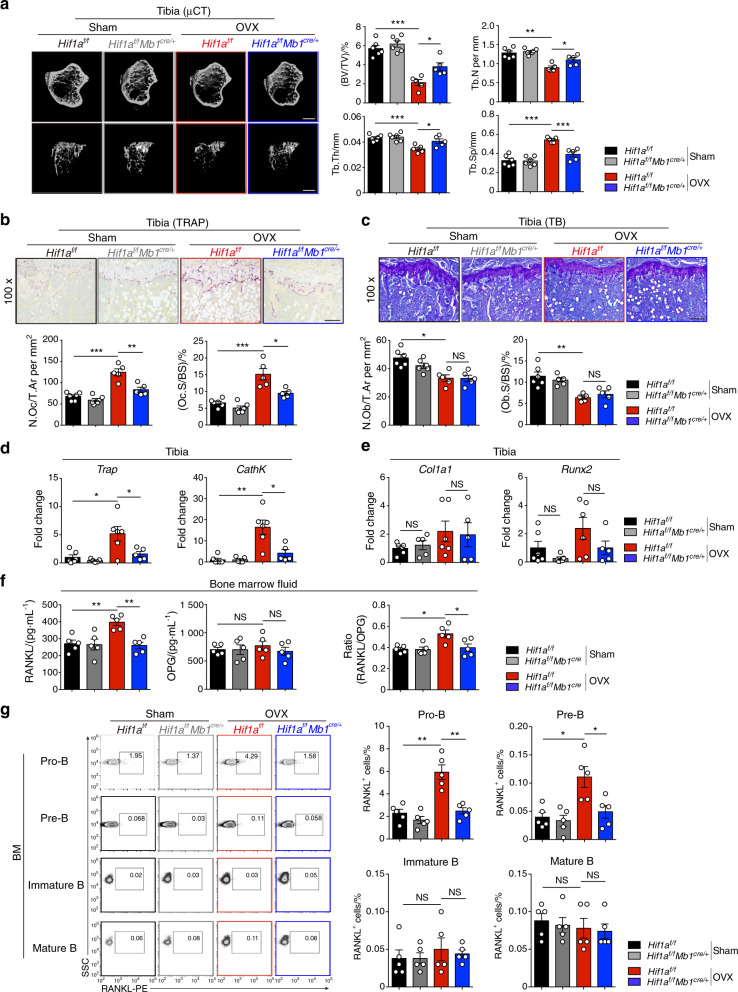
Loss of HIF-1α in B cells partially inhibits ovariectomy-induced bone loss through RANKL-mediated osteoclastogenesis. **a** Representative μCT images of tibial trabecular bone and structural parameters (BV/TV, Tb.N, Tb.Th, Tb.Sp) in sham-operated (Sham) and ovariectomized (OVX) *Hif1a*^f/f^*Mb1*^cre/+^ mice and *Hif1a*^f/f^ littermate control mice (*n* = 5, 6). Scale bars, 500 μm. Representative TRAP staining (**b**) and TB staining (**c**) in tibia from mice shown in (**a**). Bone resorption parameters (N.Oc/T.Ar, Oc.S/BS) and bone formation parameters (N.Ob/T.Ar, Ob.S/BS) in metaphyseal regions of the tibia were assessed by histomorphometric analyses. Scale bars, 100 μm. *Trap*, *CathK* (**d**), *Col1a1* and *Runx2* (**e**) mRNA expression in bone from mice shown in (**a**). **f** RANKL and OPG levels and the RANKL/OPG ratio in bone marrow fluid from mice shown in (**a**). **g** Representative plots and frequencies of RANKL^+^ cells in B cell subpopulations from mice as in (**a**). *P < 0.05, **P < 0.01, ***P < 0.001

Because B cell-derived RANKL is able to induce osteoclastoogenesis,^20^ we speculated that HIF-1α activation in B cells controls bone resorption during menopausal bone loss. To address our hypothesis, we performed OVX or sham surgery in *Hif1a*^f/f^*Mb1*^cre/+^ mice and littermate controls. No difference was observed in bone mass between *Hif1a*^f/f^*Mb1*^cre/+^ mice and their littermate controls in the sham-operated groups, whereas B cell-specific *Hif1a*-deficient mice were partially resistant to osteoporosis induced by OVX (Fig. 3a). We found that the bone volume fraction (BV/TV) in ovariectomized *Hif1a*^f/f^*Mb1*^cre/+^ mice was reduced by 41.77% compared to that in sham-operated *Hif1a*^f/f^*Mb1*^cre/+^ mice, and the BV/TV in ovariectomized *Hif1a*^f/f^ control mice was reduced by 62.65% compared to that in sham-operated controls. In addition, reduced osteoclast number and osteoclast-specific gene expression were found in *Hif1a*^f/f^*Mb1*^cre^ mice subjected to OVX (Fig. 3b, d). No difference in osteoblast number or osteoblast-specific gene expression between sham-operated and ovariectomized control mice when compared to the respective mutant mice was observed (Fig. 3c, e).

RANKL production by immune cells from ovariectomized or sham-operated mice was examined. Interestingly, only B cells in bone marrow, but not other immune cell populations, showed increased RANKL production after OVX surgery (Fig. S3a–d). Since RANKL expression was strikingly high in the Pro-B and Pre-B subsets (Fig. S3e, f), RANKL production was quantified in B cell subsets in *Hif1a*^f/f^*Mb1*^cre/+^ and littermate control mice. In line with the lack of RANKL expression in *Hif1a*-deficient B cells in vitro (Fig. 2), RANKL levels in bone marrow fluid and Pro-B/Pre-B cells were decreased in ovariectomized *Hif1a*^f/f^*Mb1*^cre/+^ mice compared to littermate mice, whereas OPG levels were not affected (Fig. 3f, g) (Fig. S3g). Thus, all these results imply that HIF-1α signaling in B cells is required for estrogen deficiency-induced osteoporosis by regulating B cell-derived RANKL production.

### Estrogen controls HIF-1α stabilization by regulating HSP70 expression

**Fig. 4:**
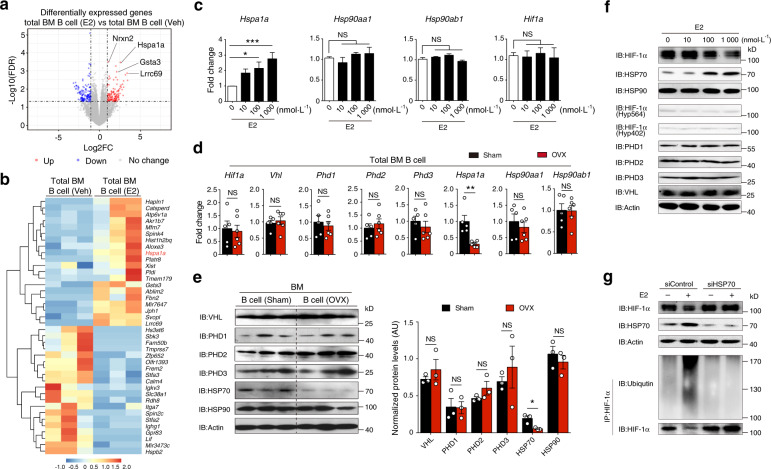
Estrogen controls HIF-1α stabilization via the HSP70-mediated degradation pathway. **a** Volcano plot of RNA-seq analysis of estrogen (E2) (1 μmol·L^**−**1^)-treated versus vehicle (Veh)-treated WT bone marrow B cells under hypoxic conditions showing genes upregulated and downregulated, respectively, with a fold change (FC) higher than 2. **b** Heatmap of differentially expressed genes found within gene sets displayed in (**a**). **c** *Hspa1a*, *Hsp90aa1*, *Hsp90ab1* and *Hif1a* mRNA expression in B cells treated with the indicated concentration of estrogen (E2) under hypoxia for 12 h (*n* = 3). Values for the control group were set as 1. **d** *Hif1a*, *Vhl*, *Phd1*, *Phd2*, *Phd3*, *Hspa1a*, *Hsp90aa1*, and *Hsp90ab1* mRNA expression in B cells from the bone marrow of sham control (Sham) and ovariectomized (OVX) mice. **e** Levels of VHL, PHD1, PHD2, PHD3, HSP70, and HSP90 protein in isolated B cells from the bone marrow of OVX and Sham control mice (*n* = 3). **f** HIF-1α, HSP70, HSP90, hydroxylated HIF-1α (P564 and P402 sites), PHD1, PHD2, PHD3, VHL and β-actin expression in isolated bone marrow B cells with the indicated concentration of estrogen (E2) treatment under hypoxic culture for 24 h. **g** HIF-1α, HSP70 and β-actin expression in bone marrow B cells transfected with control or HSP70 siRNA lentivirus under hypoxic culture for 24 h. MG132 (100 μmol·L^**−**1^) was added to HIF-1α immunoprecipitation samples. *P < 0.05, **P < 0.01, ***P < 0.001

To explore the molecular mechanism involved in abnormal activation of HIF-1α signaling during OVX-induced osteoporosis, we dissected the transcriptional network in total bone marrow B cells treated with estrogen (1 μmol·L^**−**1^) or vehicle by RNA-seq. Our analysis revealed that HSP70 (*Hspa1a*), which is responsible for protein degradation,^27^ was upregulated in estrogen-treated B cells (Fig. 4a, b). Moreover, enriched bone marrow B cells were cultured with estrogen under hypoxic conditions. Under this condition, only *Hspa1a*, but not *Hsp90aa/b1* or *Hif1a*, was induced by estrogen in a dose-dependent manner (Fig. 4c), suggesting that estrogen-induced HSP70 expression may regulate HIF-1α protein.

We also investigated the relevance of hypoxia to HIF-1α protein stabilization within the osteoporotic bone marrow niche during OVX-induced osteoporosis. The expression levels of enzymes involved in the oxygen-dependent HIF-1α degradation pathway, such as VHL, PHD1, PHD2, and PHD3, were identical in B cells from the bone marrow of OVX and sham control mice (Fig. 4d, e). When investigating the HSP pathway, which mediates oxygen-independent HIF-1α protein degradation, we found that the mRNA and protein levels of HSP70 (*Hspa1a*), but not HSP90 (*Hsp90aa/b1*), were reduced in B cells isolated from estrogen-deficient mice (Fig. 4d, e). Since HSP70 expression is regulated by heat shock factors (HSFs),^33^ we examined *Hsf1* and *Hsf2* mRNA induction and found increased *Hsf1* gene transcription in estrogen-treated B cells (Fig. S4a). In addition, three putative estrogen-responsive elements (EREs) were found on the *Hsf1* promoter using the JASPAR database (Fig. S4b). By chromatin immunoprecipitation (ChIP) assay, we showed that ERα binds to the ERE I and ERE II regions of the *Hsf1* promoter (Fig. S4c). Moreover, HSP70 expression was reduced in B cells when the *Hsf1* gene was knocked down, indicating that estrogen-induced HSP70 expression is dependent on HSF1 expression (Fig. S4d). Thus, the binding of estrogen to the ERα activates *Hsf1* transcription and HSP70 expression.

To delineate the pathway of estrogen-induced HIF-1α degradation, total bone marrow B cells were incubated with estrogen under hypoxia. Notably, reduced HIF-1α protein levels and hypoxia-related gene expression were observed in estrogen-treated B cells (Fig. 4f) (Fig. S5a). In addition, the levels of VHL, PHD1, PHD2, PHD3, and HIF-1α hydroxylation showed no difference in estrogen-treated B cells relative to control B cells (Fig. 4f). Enhanced HIF-1α ubiquitination and HIF-1α degradation induced by estrogen in B cells were abolished when HSP70 was knocked down by siHSP70 lentivirus (Fig. 4g), indicating that HIF-1α protein stability regulated by estrogen occurs in an HSP70-dependent manner. Furthermore, HSP70 overexpression led to increased HIF-1α ubiquitination and reduced hypoxia-related gene expression (Fig. S5b, c). Altogether, our data suggest that estrogen controls HIF-1α stabilization via HSP70-mediated degradation.

### The HSP70/HIF-1α axis controls RANKL production in B cells and modulates osteoclastogenesis

**Fig. 5:**
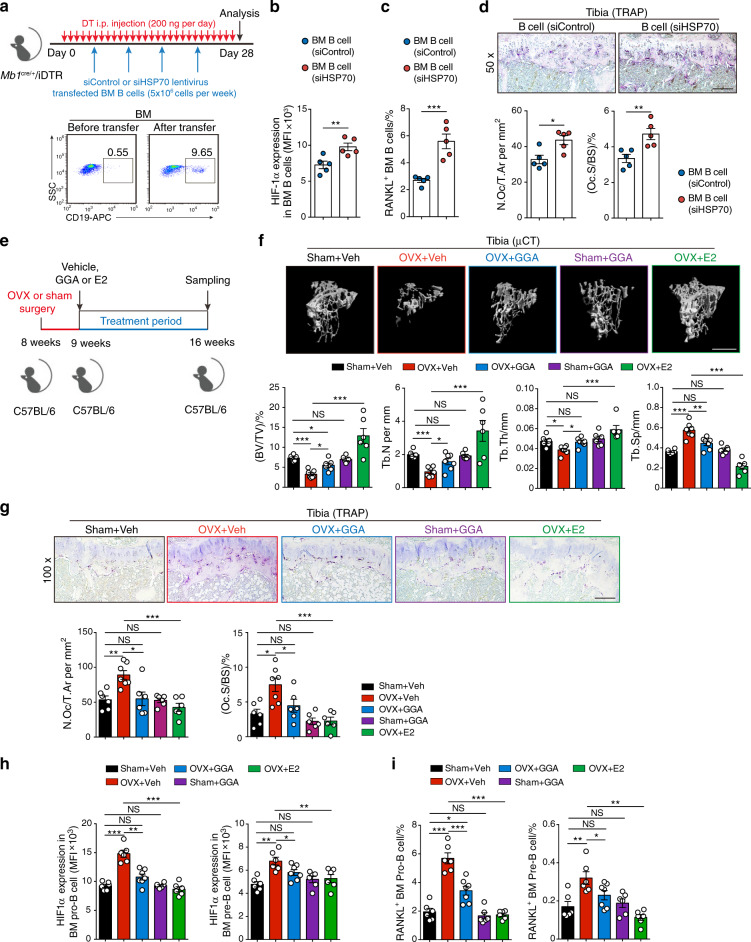
The HSP70/HIF-1α axis controls RANKL production in B cells and modulates osteoclastogenesis. **a** Scheme of adoptive transfer of siControl or siHSP70 lentivirus-transfected bone marrow B cells (5 × 10^6^ cells per week) into female recipient *Mb1*^cre/+^/iDTR mice treated daily i.p. injection of DT (200 ng·d^−1^) for 28 days. **b** HIF-1α expression in B cells from the bone marrow of *Mb1*^cre/+^/iDTR recipient mice. **c** Frequencies of RANKL^+^ B cells from the bone marrow of *Mb1*^cre/+^/iDTR recipient mice. **d** Representative TRAP staining and histomorphometry of N.Oc/T.Ar, Oc.S/BS were analyzed in tibia from mice shown in (**b**). Scale bars, 200 μm. **e** Experimental scheme of pharmacological induction of HSP70 by GGA in osteoporotic mice. **f** Representative μCT images of tibial trabecular bone and structural parameters (BV/TV, Tb.N, Tb.Th, Tb.Sp) in mice post sham surgery with vehicle (Veh) or GGA treatment, as well as OVX surgery with vehicle (Veh), GGA or estrogen (E2) treatment (*n* = 6, 7). Scale bars, 500 μm. **g** Representative TRAP staining and bone resorption parameters (N.Oc/T.Ar, Oc.S/BS) were analyzed in tibia from mice shown in (**f**) Scale bars, 100 μm. **h** HIF-1α expression in Pro-B and Pre-B populations from mice shown in (f). **i** Frequencies of RANKL^+^ Pro-B and Pre-B populations from mice shown in (**f**). *P < 0.05, **P < 0.01, ***P < 0.001

To investigate the effect of HSP70 expression on HIF-1α signaling and RANKL production in vivo, we crossed *Mb1*^cre/+^ mice with the Cre-inducible diphtheria toxin receptor (iDTR) mouse strain, resulting in mice with B cells expressing the DTR.^34^ Systemic B cell depletion was observed in *Mb1*^cre/+^/iDTR mice with daily DT injection (200 ng·d^−1^), and adoptive transfer of bone marrow B cells transfected with siHSP70 lentivirus or control lentivirus was performed in *Mb1*^cre/+^/iDTR recipient mice (Fig. 5a). In WT mice, DT injections did not alter the trabecular bone volume or osteoclast or osteoblast formation during the whole experimental process (Fig. S6a–c). Interestingly, enhanced HIF-1α expression and RANKL production were observed in HSP70-deficient B cells relative to control B cells in recipient mice (Fig. 5b, c). In addition, mice transferred with HSP70-deficient B cells displayed increased osteoclast numbers and surfaces compared to control mice (Fig. 5d), suggesting that HSP70 deficiency in bone marrow B cells enhances HIF-1α signaling activation and RANKL-mediated osteoclastogenesis.

Geranylgeranylacetone (GGA) has been shown to enhance HSP70 expression in vitro and in vivo.^35–37^ After treatment of WT mice with GGA (400 mg·kg^−1^) by oral administration, HSP70 (*Hspa1a*) gene expression was significantly increased (Fig. S6d). To further investigate whether GGA treatment can inhibit bone loss, C57BL/6 WT mice that received OVX or sham surgery were treated with vehicle, GGA or estrogen as described in Fig. 5e. Treatment with GGA attenuated OVX-induced osteopenia, increasing BV/TV and trabecular numbers in the tibia (Fig. 5f). In sham-operated mice, GGA treatment had no detectable effects on bone architecture (Fig. 5f). Reduced osteoclast numbers and surfaces were observed in GGA-treated ovariectomized mice compared to vehicle-treated ovariectomized mice (Fig. 5g). Moreover, intracellular staining of HIF-1α and RANKL in the Pro-B and Pre-B populations showed low-level expression after GGA treatment (Fig. 5h, i), suggesting that GGA-induced HSP70 expression suppresses HIF-1α signaling activation and consequently RANKL production. Thus, the HSP70/HIF-1α axis in B cells controls RANKL-mediated osteoclastogenesis in vivo.

### *RANKL* gene expression correlates with *HIF1A* gene expression in human B cells

**Fig. 6:**
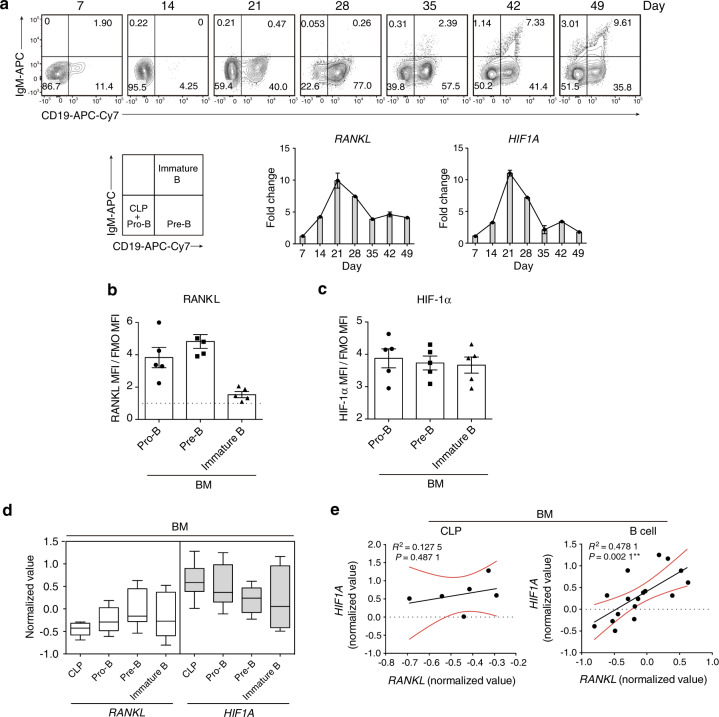
*RANKL* gene expression is associated with *HIF1A* gene expression in human B cells. **a** Progressive development of cord blood-derived human CD34^+^ cells under B cell differentiation conditions for 49 days. *RANKL* and *HIF1A* gene expression was examined at the indicated time points (*n* = 3). RANKL (**b**) and HIF-1α (**c**) expression in human CD34^+^ cell-derived Pro-B, Pre-B, and immature B populations. **d** Normalized expression values of *RANKL* and *HIF1A* in human CLP, Pro-B, Pre-B, and immature B populations from the public gene expression database GSE14714. Bounds of boxes and whiskers represent the min-to-max normalized value of gene expression in each population. Medians are indicated in each box as centerline. **e** Correlation between *HIF1A* and *RANKL* expression values in human CLP and bone marrow B cells from the public gene expression database GSE14714 (*R*^2^ and *P* values are indicated). **P < 0.01 by Pearson’s test (**e**)

We also investigated HIF-1α and RANKL expression during human B lymphopoiesis. Cord blood-derived CD34^+^ cells were cultivated under B-cell differentiation conditions as previously described^38^ and progressively developed into Pro-B, Pre-B, and immature B cells (Fig. 6a). We observed increased mRNA expression of *HIF1A* and *RANKL* up to day 21, when the culture was enriched in the Pro-B and Pre-B populations, which remained high at day 28 when cells were mostly Pre-B cells (Fig. 6a). As mRNA analysis of the culture cannot distinguish the individual expression within a specific population, we studied the expression of HIF-1α and RANKL protein by flow cytometry. The Pro-B and Pre-B populations displayed the expression of both RANKL and HIF-1α (Fig. 6b, c). To further define whether *RANKL* expression correlates with *HIF1A* expression in human B cells, *RANKL* and *HIF1A* mRNA expression profiles in the public gene expression database GSE14714 were analyzed. Similar *RANKL* and *HIF1A* mRNA expression was observed in bone marrow B cell subsets during B cell differentiation in vivo (Fig. 6d). Interestingly, a positive correlation between *RANKL* and *HIF1A* expression was observed in B cells but not in CLP cells (Fig. 6e).

With the evidence of the HSP70 inducer GGA as a drug improving bone resorption in a murine OVX model, we also investigated the potential association between *HSP70*, *RANKL*, or *HIF1A* gene expression and bone mineral density in postmenopausal patients. To this end, we used a set of publicly available genomics data deposited in GSE (accession no. GSE7429), where DNA microarray experiments were performed using circulating B cells obtained from 10 postmenopausal women.^39^ In this dataset, the *HSP70 (HSPA1A)* gene expression level was positively associated with the BMD Z-score, suggesting that HSP70 might be a potent therapeutic target for osteoporosis (Fig. S7).

## Discussion

**Fig. 7:**
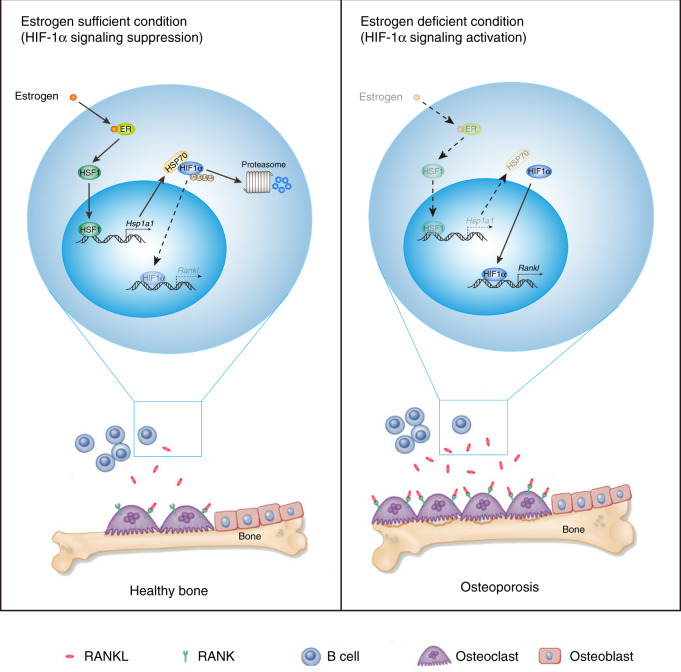
HIF-1α signaling in B lymphocytes regulates bone homeostasis through RANKL-mediated osteoclastogenesis.

In this article, we describe a new function of HIF-1α in bone marrow B cells. HIF-1α controls RANKL production in B cells, thereby locally enhancing osteoclast differentiation and contributing to bone metabolism. Activation of HIF-1α signaling in B cells was suppressed by estrogen-mediated HSP70 expression (Fig. 7). Accordingly, treatment with the HSP70 inducer GGA ameliorated OVX-induced bone loss.

Regulatory factors from immune cells have been implicated in many disorders of low bone mass, including postmenopausal osteoporosis, hyperparathyroidism, periodontal infection and rheumatoid arthritis.^20,40–43^ During bone loss, a marked increase in inflammatory cytokines was observed. These inflammatory cytokines enhance osteoclast precursor formation, inhibit osteoblast proliferation and enhance osteoporosis.^44^ However, the RANK/RANKL/OPG signaling pathway is the final common effector system, which regulates the equilibrium between bone formation and resorption.^19,45,46^ Pre-B leukemia cells have been shown to produce high levels of RANKL, which are sufficient to reduce bone mass in acute lymphoblastic leukemia (ALL).^47^ Our study demonstrated that Pro-B and Pre-B cells with high HIF-1α expression produce more RANKL than immature/mature B cells with low HIF-1α expression, consistent with recent findings that HIF-1α activity in bone marrow B cells varies at different developmental stages within the bone niche.^48^

Previous studies have shown that mTORC1 controls RANKL/OPG expression in B cells through inactivation of Akt and downregulation of β-catenin.^21^ However, the influence of the hypoxic microenvironment in bone marrow on B cell-derived RANKL production remains poorly understood. Herein, we demonstrated the binding of HIF-1α to the *Rankl* promoter, indicating that HIF-1α is a key transcription factor for RANKL production in B cells and subsequent osteoclastogenesis and bone resorption. Interestingly, our data show that Pro-B and Pre-B cell populations but not other immune cell populations have an increased level of RANKL production under estrogen-deficient conditions, and the different estrogen intracellular signaling in these immune cell populations may explain this increase.

Based on our findings, we propose that estrogen allows the maintenance of the HIF-1α protein at an optimal level under steady-state conditions. Under estrogen-deficient conditions, however, an excess of RANKL is produced by bone marrow B cells that show constitutively activated HIF-1α signaling. RNA-seq and ChIP-seq analysis data showed that out of 2954 HIF-1α binding genes, 22 were transcriptionally dysregulated under estrogen-deficient conditions. Among them, the most relevant gene for bone homeostasis was RANKL (*Tnfsf11*), while others (*Jup*, *Ust*, *Gnaz*, *Kitl*, *Aif1*, *Kalm*, *Mylk*, *Parvb*, *Cpt1a*, *Fgf1*, *Fgf13*, *Dlg2*, *Shank2*, *Dgki*, *Igf2bp3*, *Mmrn1*, *Ccdc148*, *Nr1h3*, *Fbn1*, *Cndb2*, *Scn4b*) were related to cytoskeletal organization, cell migration and metabolism.

HSP family members, including HSP70 and HSP90, have been studied extensively in several bone diseases, such as osteosarcomas and osteoarthritis.^49^ The lower HSP70 expression level in B cells in the context of estrogen deficiency as well as the enhanced HSP70 expression during estrogen treatment suggest that HSP70 might serve as a pivotal regulator in HIF-1α-mediated RANKL production. The acyclic isoprenoid compound GGA, a known inducer of HSP70 expression through activation of HSF1, has been used in clinical antiulcer therapy.^50^ After in vivo GGA treatment, we observed suppression of HIF-1α signaling, a decrease in RANKL production and protection against osteoporosis. These results imply that the HSP70/HIF-1α axis is a potential therapeutic target for osteoporosis disorder treatment. Interestingly, we found a positive association of *HSPA1A* gene expression in circulating B cells with BMD Z-score in postmenopausal women. However, a correlation between *RANKL* and *HIF1A* gene expression was not detectable in this dataset, likely because of the low sample numbers, sampling limitations, and low stability of *HIF1A* in the peripheral microenvironment.^51,52^ Therefore, further studies on local bone marrow B cells from postmenopausal patients will be urgently needed to understand the role of B cell-specific HIF-1α signaling in postmenopausal osteoporosis.

In summary, we provide a novel molecular mechanism for the regulation of bone loss by HIF-1α signaling in bone marrow B cells. HIF-1α effectively binds to the HRE region in the *Rankl* promoter, leading to increased RANKL production and enhanced osteoclastogenesis in a model of postmenopausal osteoporosis. By modulating HSP70-mediated protein degradation, estrogen regulates HIF-1α levels and RANKL production. Moreover, pharmacological induction of HSP70 by GGA inhibits HIF-1α activation in bone marrow B cells, suppresses osteoclastogenesis and protects against estrogen deficiency-induced low bone mass. Collectively, HIF-1α expression in B cells regulates bone homeostasis via RANKL-mediated osteoclast formation.

## Materials and methods

### Animal experiments

*Hif1a*^f/f^, *Vhl*^f/f^, and *Mb1*^cre^ mice were previously described.^53–55^ We generated B cell-specific *Hif1a* or *Vhl* knockout mice by crossing *Hif1a*^f/f^ or *Vhl*^f/f^ mice with *Mb1*^cre^ mice. *Vhl*^f/f^ or *Hif1a*^f/f^ cre-negative or *Mb1*^cre/+^ heterozygous littermates were used as control mice. Bilateral OVX or sham surgery was performed as a surgical model of menopause in eight-week-old female *Hif1a*^f/f^ and *Hif1a*^f/f^ *Mb1*^cre^ mice. For the GGA or E2 treatment experiment, OVX or sham surgery was performed in eight-week-old female mice. One week after surgery, mice were orally given GGA (400 mg·kg^−1^) every other day for seven weeks. The E2 treatment group was used as the positive control group according to this protocol.^56^ To deplete bone marrow B cells in mice, iDTR transgenic mice were crossed with *Mb1*^cre^ mice. Ten-week-old female *Mb1*^cre/+^/iDTR mice were injected with 200 ng diphtheria toxin daily as previously described.^34^ Enriched CD19^+^ bone marrow B cells were transfected with siControl or siHSP70 plasmid lentiviral particles, and a total of five million transduced B cells were adoptively transferred into *Mb1*^cre/+^/iDTR mice once a week for four weeks. All mice were maintained at the animal facility of Friedrich-Alexander-Universität Erlangen-Nürnberg Faculty of Medicine, and experiments were approved by the Regierung von Mittelfranken ethics committee (55.2-2532-2-678).

### Flow cytometry

Single-cell suspensions were prepared and stained with the following antibodies: anti-CD4 (1:800), anti-CD8 (1:800), anti-CD45 (1:500), anti-CD11b (1:400), anti-Ly6G (1:400), anti-F4/80 (1:400), anti-IgD (1:200), anti-CD19 (1:400), anti-CD25 (1:200), anti-RANKL (1:100), anti-CD43 (1:200), anti-B220 (1:400), anti-IgM (1:200) and anti-HIF-1α (1:50). Gating strategies are included in Fig. S8.

### μCT analysis

The proximal metaphyses of the tibiae were scanned by a μCT 40 system (Scanco Medical AG, Switzerland) using previously described parameters.^57^ Segmentation of 3D volumes was conducted using the predefined script Open VMS from Scanco.

### RANKL and OPG measurements

Bone marrow fluid was collected as described previously.^58^ RANKL and OPG levels were measured by a DuoSet ELISA Development kit (R&D).

### Lentivirus transfection

Lentivirus preparation and B cell transduction were described previously.^59^ Briefly, 293T cells were cotransfected with psPax2 plasmid (Addgene), VSVG plasmid (Addgene), siControl plasmid, siHSP70 plasmid or siHSF1 plasmid (ABM, Richmond, BC, Canada), lenti-EV (empty vector) or lenti-HSP70 overexpression plasmid (Sinobiological) using Lipofectamine 2000 (Thermo Fisher). Supernatants were collected and filtered. Isolated bone marrow B cells were transduced by lentiviral particles by spin infection.

### Western blot and immunoprecipitation

Western blotting and immunoprecipitation were performed using previous methods.^60^ HIF-1α antibody (1:1 000, 10006421; Cayman), VHL antibody (1:1 000, sc-5575; Santa Cruz), PHD1 antibody (1:1 000, NB100-310; Novus), PHD2 antibody (1:1 000, NB100-2219; Novus), PHD3 antibody (1:1 000, NB100-303; Novus), HSP70 antibody (1:500, ADI-SPA-812; Enzolife), HSP90 antibody (1:500, sc-13119; Santa Cruz) and β-actin antibody (1:2 000, A2066; Sigma) were used for immunoblotting. Protein bands were quantified using ImageJ. For immunoprecipitation, nuclear extracts from cultured B cells were incubated with Dynabeads protein G and 5 μg anti-HIF-1α antibody (H1a67) overnight at 4 °C. Anti-HIF-1α, (1:1 000, 10006421; Cayman) anti-HSP70 (1:500, ADI-SPA-812; Enzolife) and anti-ubiquitin (1:500, sc-166553; Santa Cruz) antibodies were used for immunoblotting. Western blot source data are included in Fig. S9.

### Immunofluorescence, histology and histomorphometry

For immunofluorescence staining, sections were incubated with anti-HIF-1α antibody (1:50, H1a67, Novus) and anti-B220 antibody (1:100, RA3-6B2, Abcam) followed by incubation with AF488- and AF647-conjugated secondary antibodies (1:200, VECTOR). Fluorescence images were captured by a Zeiss confocal microscope. For histological analysis, serial paraffin sections were stained using an H&E staining kit (Carl Roth), Leukocyte Acid Phosphatase Kit (Sigma) or Toluidine Blue. For histomorphometry evaluation, a Nikon microscope equipped with a histomorphometry analysis system (OsteoMeasure; Osteometrics) was used. All samples were blinded for quantitative analysis.

### RNA sequencing and ChIP sequencing

RNA samples were prepared using RNA-Solv Reagent (Omega Bio-tek) or RNeasy kit (QIAGEN), and RNA sequencing was performed by Novogene (London, UK). RNA-seq data in this study were deposited in the GEO database with the accession IDs GSE163704, GSE163848, and GSE163849. ChIP experiments were performed using a ChIP-IT kit (Active Motif) followed by high-throughput Illumina sequencing. ChIP-seq data in this study were deposited in the GEO database with the accession ID GSE163704.

### Human material

The collection control BM was approved by the University Freiburg Ethics Committee (507/16). Control BM derived from otherwise healthy subjects undergoing orthopedic surgery or BM aspirate performed for exclusion of malignancy. All subjects provided informed consent. Umbilical cord blood (CB) was obtained with the approval of the University Freiburg Ethics Committee (353/07_120590).

### CD34^+^ cell isolation and culture

CD34^+^ cells were enriched from human CB or bone marrow using a CD34 MicroBead Kit (Miltenyi Biotec). CD34^+^ cells were cultivated in 96-well plates at 10^5^ cells per mL in Iscove’s medium with the addition of human IL-6, stem cell factor (SCF), and Flt3-L (25 ng·mL^**−**1^, Immunotools). After 7 days, cells were harvested and replated at 1 × 10^5^ cells per mL and incubated with human IL-7 (20 ng·mL^**−**1^, Immunotools), SCF and Flt3-L (25 ng·mL^**−**1^, Immunotools).

### Microarray datasets analysis

In the current study, we used gene expression (GSE14714 and GSE7429) datasets from NCBI. Sorted CLP, Pro-B, Pre-B, and immature B populations from six different healthy volunteers were enrolled in the GSE14714 program, while sorted circulating B cells in whole blood from ten low BMD patients were enrolled in the GSE7429 program.

### Statistical analysis

Two-tailed Student’s *t*-test were performed for comparisons between two groups. One-way analysis of variance (ANOVA) or two-way ANOVA was used for comparisons among multiple groups. Pearson’s test was performed for correlation. *P* < 0.05 was considered statistically significant. GraphPad Prism software 6.0 was used for all statistical analyses.

## Supplementary information

supplemental Information

## Data Availability

Data and materials will be made available upon request and, if applicable, material transfer agreements.
