# Supplementary material for: Estrogen-mediated downregulation of HIF-1α signaling in B lymphocytes influences postmenopausal bone loss
Source: Bone Res. 2022 Feb 17;10:15. doi: 10.1038/s41413-022-00189-x (PMC8854586; doi:10.1038/s41413-022-00189-x)
Supplement: Supplementary file 1 — supplemental Information [file 41413_2022_189_MOESM1_ESM.docx]

**Supplementary Information**

Supplementary methods

Supplementary Fig 1. HIF-1α signaling and estrogen intracellular signaling in immune cells from bone marrow, spleen and draining lymph nodes.

Supplementary Fig 2. *Mb1*-cre specificity and western blot analysis of HIF-1α, VHL expression in isolated bone marrow B cells from *Mb1*^cre/+^, *Vhl*^f/f^*Mb1*^cre/+^ and *Vhl*^f/f^*Hif1a*^f/f^*Mb1*^cre/+^ mice.

Supplementary Fig 3. RANKL expression in immune cell subsets from bone marrow, spleen and draining lymph nodes.

Supplementary Fig 4. Estrogen regulates HSP70 expression via transcriptional factor HSF1.

Supplementary Fig 5. Impact of estrogen treatment or HSP70 overexpression in bone marrow B cells on hypoxia-related gene expression.

Supplementary Fig 6. Impact of DT treatment on trabecular bone volume, osteoclast and osteoblast formation.

Supplementary Fig 7. The correlation of gene expression or body mass index with Spine Z-score in circulating B cells from blood of postmenopausal osteoporosis patients.

Supplementary Fig 8. FACS gating strategies.

Supplementary Fig 9. Western blot source data.

Supplementary Table 1. List of HIF-1α targeted gene name and function.

Supplementary Table 2. List of primers for qPCR, ChIP and subcloning.

**Supplementary methods**

**Animal Experiments**

Ai14 tdTomato reporter mice were kindly provided by Prof. Gerhard Krönke. To address *Mb1*-cre specificity, *Mb1*-cre mice were crossed with Ai14 tdTomato reporter mice. tdTomato expression was examined by cytometry in bone marrow B cell, CD4 T cell, CD8 T cell and myeloid cell from 6-weeks-old *Mb1*^cre/+^/tdTomato mice or control tdTomato mice. To test the side effect of DT injection on bone homeostasis, 8-week-old female C57BL/6 mice were i.p injected with 200 ng DT daily for 28 days.

**Bone marrow B cells isolation and culture**

For B cells isolation, resting bone marrow B cells were purified by anti-CD19 magnetic beads (MiltenyiBiotech) following the manufacturer’s instructions. The purified B cells population was > 95% CD19 positive cells. B cells were cultured in RPMI supplemented with 10% FCS, β-ME, penicillin (100 U/ml) and streptomycin (100 μg/ml) under normoxia (21% O_2_) or hypoxia (1% O_2_).

**Quantitative PCR analysis**

Total cell or tissue RNA was extracted using Trizol reagent (Invitrogen) and complementary DNA was synthesized by using High-Capacity cDNA Reverse Transcription Kit (Thermo Scientific) according to the manufacturer’s instructions. Quantitative PCR (QPCR) were performed using SYBR Green I-dTTP (Eurogentec). Specific primers used for QPCR are listed in Supplementary Table 2. The levels of gene mRNA expression were determined by evaluating the threshold cycle (Ct) of target gene after normalization against the Ct value of *Hprt* and calculated using the formula 2^-(Ct of target gene-Ct of^ *^Hprt^*^)^.

**Luciferase reporter assay**

HRE regions (I, II) of *Rankl* promoter were amplified by PCR from genomic DNA extracted from splenocytes in C57BL/6 WT mice and cloned into the pGL3 firefly reporter vector (Promega). CH12F3 cells were co-transfected with luciferase reporter construct and β-gal plasmid using electroporation. Transfected cells were cultured under normoxic (21% O_2_) or hypoxic (1% O_2_) conditions for 24 h. Cells were then lysed and luciferase activity was quantified and normalized to the activity of the co-transfected β-gal reporter gene.

**Chromatin immunoprecipitation (ChIP)**

For HIF-1α ChIP experiment, isolated bone marrow B cells were enriched from C57BL/6 WT mice and cultured in normoxia (21% O_2_) or hypoxia (1% O_2_) for 12 h. Next, chromatin immunoprecipitation experiments were performed with ChIP-IT Express kit (Active Motif) according to the manufacturer’s protocol. 10 μg anti-HIF-1α antibodies and control IgG, were used for the immunoprecipitation. For ERα ChIP experiment, isolated bone marrow B cells were enriched from C57BL/6 WT mice and cultured with or without estrogen treatment for 24 h. Next, chromatin immunoprecipitation experiments were performed with ChIP-IT Express kit (Active Motif) according to the manufacturer’s protocol. 10 μg anti-ERα antibodies and control IgG, were used for the immunoprecipitation. Primer sequence for HIF-1α and ERα binding site were shown in Supplementary Table 2.

**Supplementary Fig 1. HIF-1α signaling and estrogen intracellular signaling in immune cells from bone marrow, spleen and draining lymph nodes.**

**a** Representative plots and quantification of HIF-1α expression in CLP (Lin^-^IL7Ra^+^Sca1^+^cKit^+^) from sham-operated and ovariectomized mice (n=10 per group). MFI, mean fluorescence intensity. **b** Quantification of HIF-1α expression in CLP and Pro-B cells from sham-operated and ovariectomized mice (n=10 per group). MFI, mean fluorescence intensity. **c** Heatmap showing the relative expression levels of key HIF-1α target genes in bone marrow B cells from sham-operated and ovariectomized mice (n=3 per group). **d** Representative plots and quantification of HIF-1α expression in immature B (B220^mid^IgD^-^IgM^+^) and mature B (B220^hi^IgD^+^IgM^+^) cells from sham-operated and ovariectomized mice (n=5 per group). MFI, mean fluorescence intensity. **e** Representative plots and quantification of HIF-1α expression in B cells from spleen (SP) and draining lymph node (dLN) of sham-operated and ovariectomized mice (n=10 per group). MFI, mean fluorescence intensity. **f** Heatmap showing the relative expression levels of key estrogen intracellular signaling genes in bone marrow Pro-B, Pre-B, Immature B, Mature B, Neutrophils, Monocytes, CD4 and CD8 T cells. **g** Quantification of estrogen receptor α (ERα) expression in bone marrow B cells, Neutrophils, Monocytes, CD4 and CD8 T cells from sham-operated and ovariectomized mice (n=8 per group). MFI, mean fluorescence intensity. **h** Quantification of ERα expression in bone marrow B subsets from WT mice (n=3 per group). MFI, mean fluorescence intensity. Pictures are representative of three independent experiments. Data are shown as mean ± s.e.m. NS, not significant and * *P* < 0.05, ** *P* < 0.01 by Student’s *t*-test.

**Supplementary Fig 2. *Mb1*^cre^ specificity and western blot analysis of HIF-1α and VHL expression in isolated bone marrow B cells from *Mb1*^cre/+^, *Vhl*^f/f^*Mb1*^cre/+^ and *Vhl*^f/f^*Hif1a*^f/f^*Mb1*^cre/+^ mice.**

**a** Diagram of the loxP-flanked STOP cassette upstream of tdTomato with Cre recombination. **b** Representative plots of tdTomato expression in bone marrow B cell, CD4 T cell, CD8 T cell and myeloid cell from *Mb1*^cre^/tdTomato reporter mice. **c** HIF-1α and VHL protein expression in isolated bone marrow B cells from *Mb1*^cre/+^, *Vhl*^f/f^*Mb1*^cre/+^ and *Vhl*^f/f^*Hif1a*^f/f^*Mb1*^cre/+^ mice.

**Supplementary Fig 3. RANKL expression in immune cell subsets from bone marrow, spleen and draining lymph nodes.**

**a** Representative plots and frequencies of total RANKL expression in bone marrow CLP, B cells, Neutrophils, Monocytes, CD4 and CD8 T cells from sham-operated and ovariectomized mice (n=6 or 10 per group). **b** Absolute number of RANKL^+^ bone marrow CLP, B cells, Neutrophils, Monocytes, CD4 and CD8 T cells from mice shown in (a). **c** Quantification of RANKL expression in CLP and Pro-B cells from sham-operated and ovariectomized mice (n=10 per group). MFI, mean fluorescence intensity. **d** Representative plots and quantification of RANKL expression in B cells from spleen (SP) and draining lymph node (dLN) of sham-operated and ovariectomized mice (n=10 per group). MFI, mean fluorescence intensity. **e** Representative plots and frequencies of total RANKL expression in Pro-B, Pre-B, immature and mature B cells (n=5 per group). **f** Representative plots and frequencies of surface RANKL expression in Pro-B, Pre-B, immature and mature B cells (n=5 per group). **g** Representative plots and frequencies of surface RANKL expression in Pro-B, Pre-B, Immature B and Mature B cells from sham-operated and ovariectomized mice (n=5 per group). Pictures are representative of three independent experiments. Data are shown as mean ± s.e.m. NS, not significant; * *P* < 0.05; ** *P* < 0.01 by Student’s *t*-test (a-c) or one-way ANOVA (g) with Bonferroni’s multiple comparison post hoc test.

**Supplementary Fig 4. Estrogen regulates HSP70 expression via transcriptional factor HSF1.**

**a** Quantitative RT-PCR analysis of *Hsf1* and *Hsf2* expression in bone marrow B cells from WT mice after treatment with increased concentration of estrogen (E2) under hypoxia for 12 h (n=3 per group). Values for control group were set as 1. **b** Schematic analysis of estrogen response elements (EREs) on *Hsf1* promoter. **c** ChIP assays showing the recruitment of the endogenous estrogen receptor α (ERα) on the ERE regions of *Hsf1* promoter in bone marrow B cells with vehicle or estrogen treatment for 12 h (n=3 per group). **d** Western blot analysis of HSF1, HSP70 and β-actin in isolated bone marrow B cells transfected with control or HSF1 siRNA lentivirus under hypoxia for 24 h. Pictures are representative of three independent experiments. Data are shown as mean ± s.e.m. * *P* < 0.05, *** *P* < 0.001 by one-way ANOVA (a) or two-way ANOVA (c) with Bonferroni’s multiple comparison post hoc test.

**Supplementary Fig 5. Impact of estrogen treatment or HSP70 overexpression in bone marrow B cells on hypoxia-related gene expression.**

**a** Heatmap of hypoxia-related gene expression in estrogen (E2) (1 μM)-treated (n=3) or vehicle (Veh)-treated (n=3 per group) WT bone marrow B cells under normoxic or hypoxic condition for 24 h. **b** Western blot analysis of HIF-1α, HSP70 and β-actin in isolated bone marrow B cells transfected with empty vector lentivirus (lenti-EV) or HSP70 overexpression lentivirus (Lenti-HSP70) under hypoxia for 24 h. 100 μM MG132 was added for the final 4 h in HIF-1α immunoprecipitation samples. Ubiquitinated HIF-1α, HIF-1α and HSP70 are shown. **c** Heatmap of hypoxia-related gene expression in empty vector lentivirus (lenti-EV) or HSP70 overexpression lentivirus (Lenti-HSP70) transfected WT bone marrow B cells (n=4 per group) under normoxia or hypoxic condition for 24 h.

**Supplementary Fig 6. Impact of DT treatment on trabecular bone volume, osteoclast and osteoblast formation.**

**a** Representative H&E staining in tibias from mice treated with daily i.p injection of DT (200 ng/day) or vehicle PBS and quantification of trabecular bone fraction (BV/TV), trabecular number (Tb.N) were assessed by bone histomorphometric analyses of the metaphyseal regions of tibias. Scale bars, 100 μm. **b** and **c** Representative TRAP staining (b) and TB staining (c) in tibias from mice shown in (a). Number of osteoclastic cells per tissue area (N.Oc/T.Ar), osteoclast surface normalized by bone surface (Oc.S/BS), number of osteoblastic cells per tissue area (N.Ob/T.Ar) and osteoblast surface normalized by bone surface (Ob.S/BS), were assessed by bone histomorphometric analyses of the metaphyseal regions of tibias. Scale bars, 100 μm. **d** *Hsp1a1*, *Hsp90aa1* and *Hsp90ab1* gene expression in enriched bone marrow B cells after GGA treatment (n=3 per group). Values of *Hsp1a1* for control group were set as 1. Pictures are representative of three independent experiments. Data are shown as mean ± s.e.m. *** *P* < 0.001 by two-way ANOVA with Bonferroni’s multiple comparison post hoc test.

**Supplementary Fig. 7 The correlation of gene expression or body mass index with Spine Z-score in circulating B cells from blood of postmenopausal osteoporosis patients.**

**a** Correlation between clinical spine Z-score and *HSP1A1*, *HSP90AB1*, *RANKL* or *HIF1A* gene expression, *RANKL* gene expression and *HSP1A1* or *HIF1A* gene expression in circulating B cells from blood of postmenopausal osteoporosis patients (n=10) from public gene expression database (GSE7429) (R^2^ and *p* values are indicated). The linear regression line is superimposed, surrounded with the 95% confidence interval lines for the regression line. **b** Correlation of Body mass index with Spine Z-score in circulating B cells from blood of postmenopausal osteoporosis patients (R2 and *p* values are indicated). The linear regression line is superimposed, surrounded with the 95% confidence interval lines for the regression line. * *P* < 0.05 by Pearson test.

**Supplementary Fig 8. FACS gating strategies.**

**a** Gating strategies of CD4 T cells, CD8 T cells, B cells, monocytes and neutrophils in bone marrow cells. **b** Gating strategies of CLP and Pro-B cells in bone marrow cells. **c** Gating strategies of Pro-B, Pre-B, immature B and mature B cells in bone marrow cells. **d** Gating strategies of human Pro-B, Pre-B and immature B cells.

**Supplementary Fig 9. Western blot source data.**


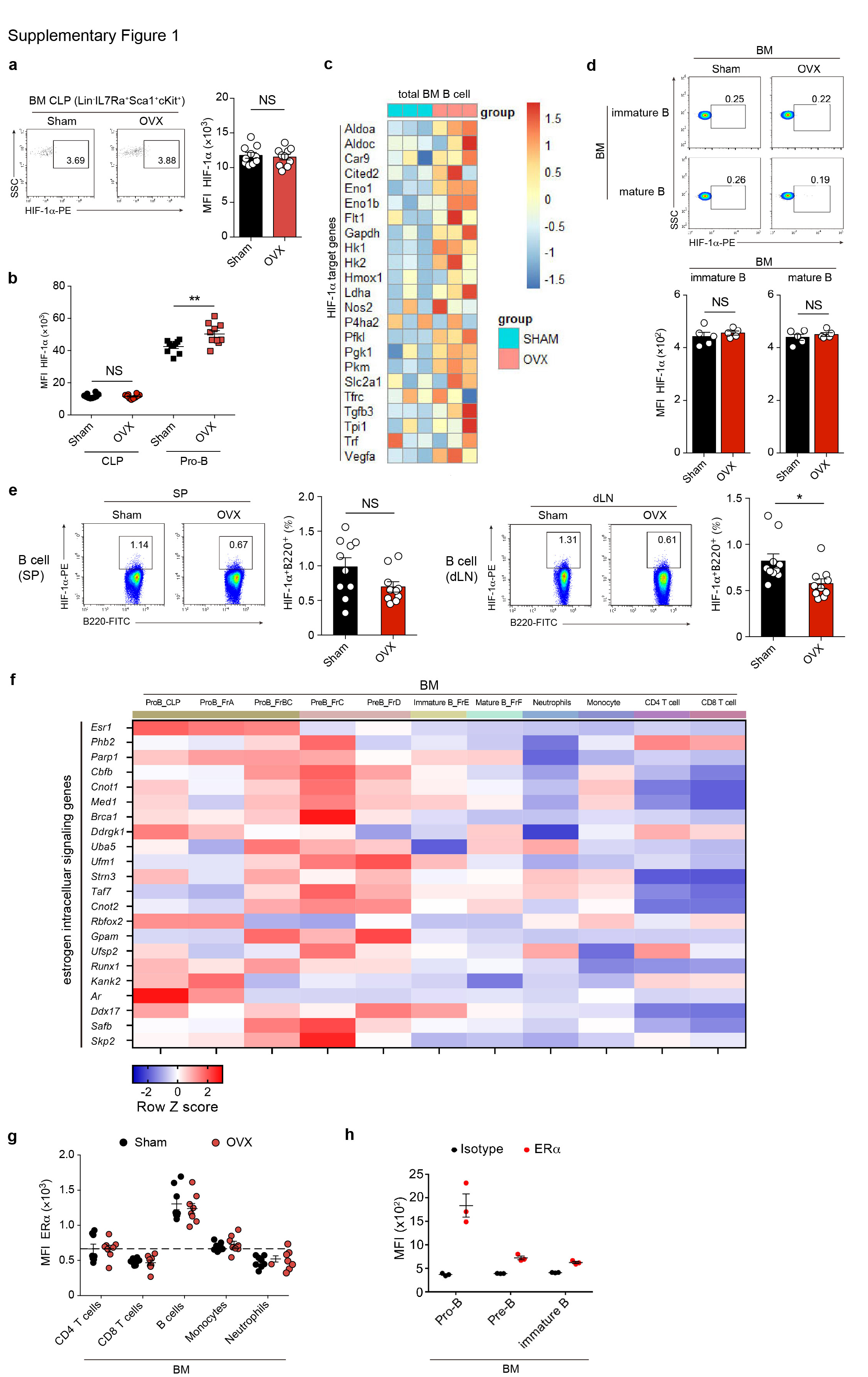


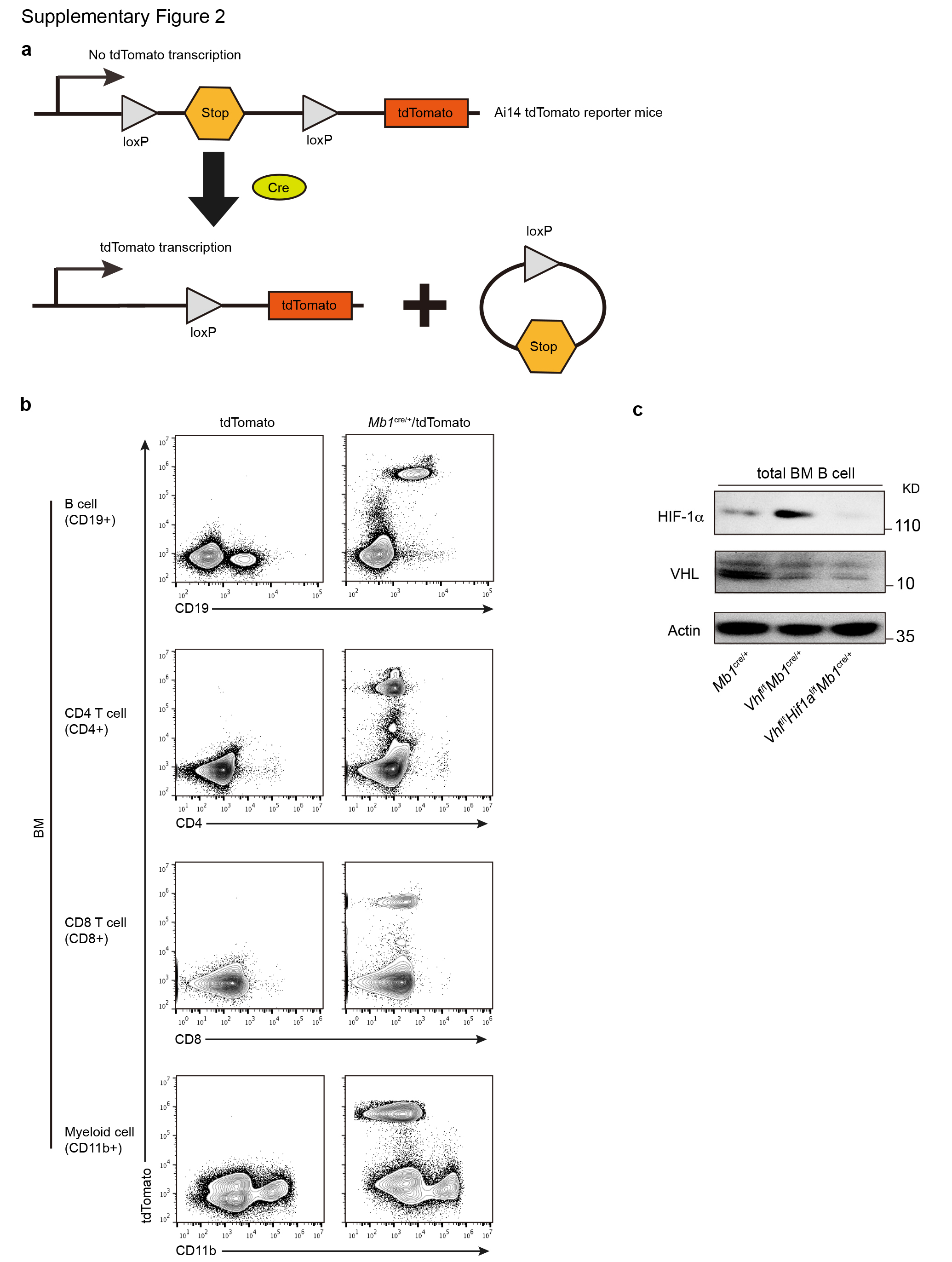


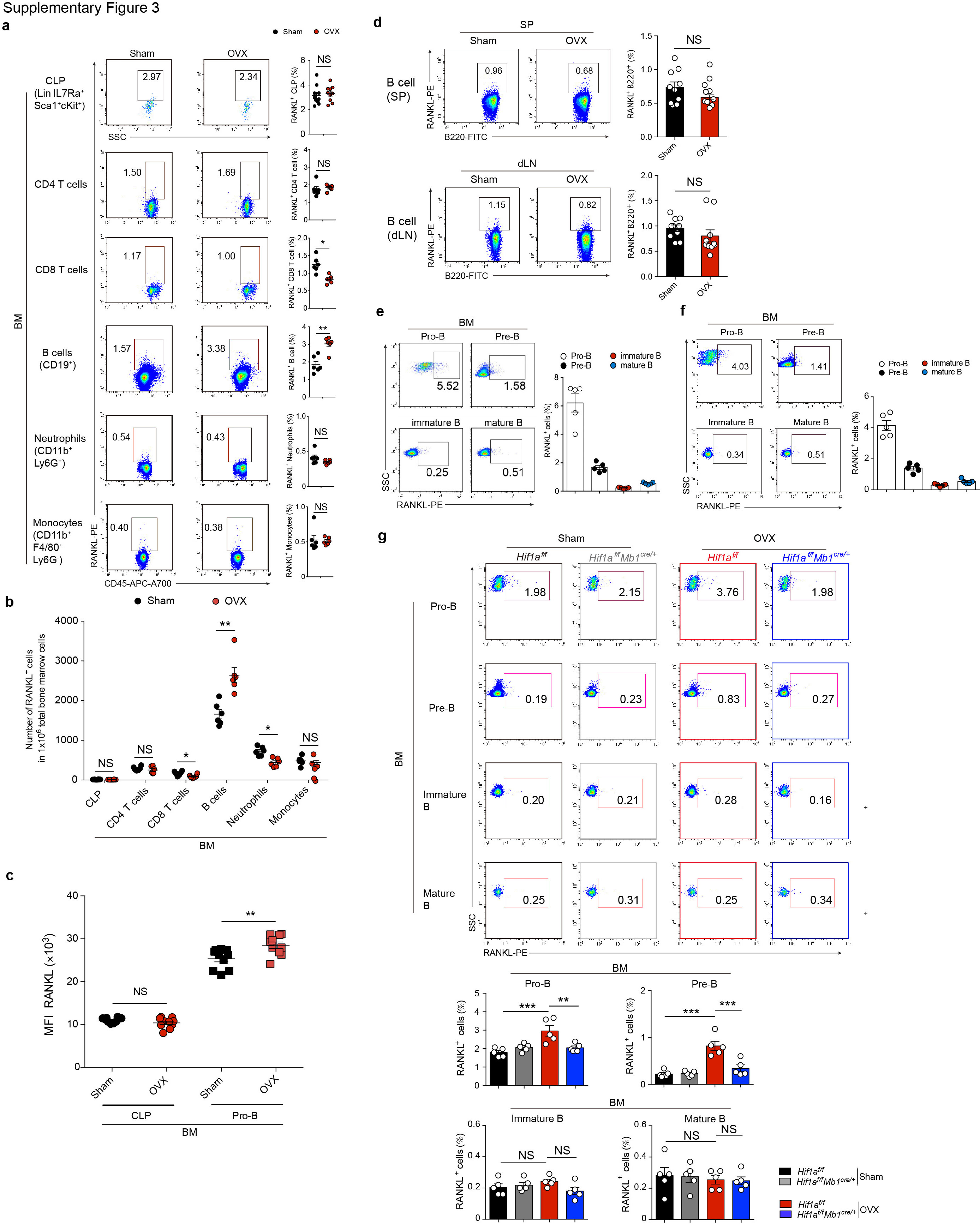


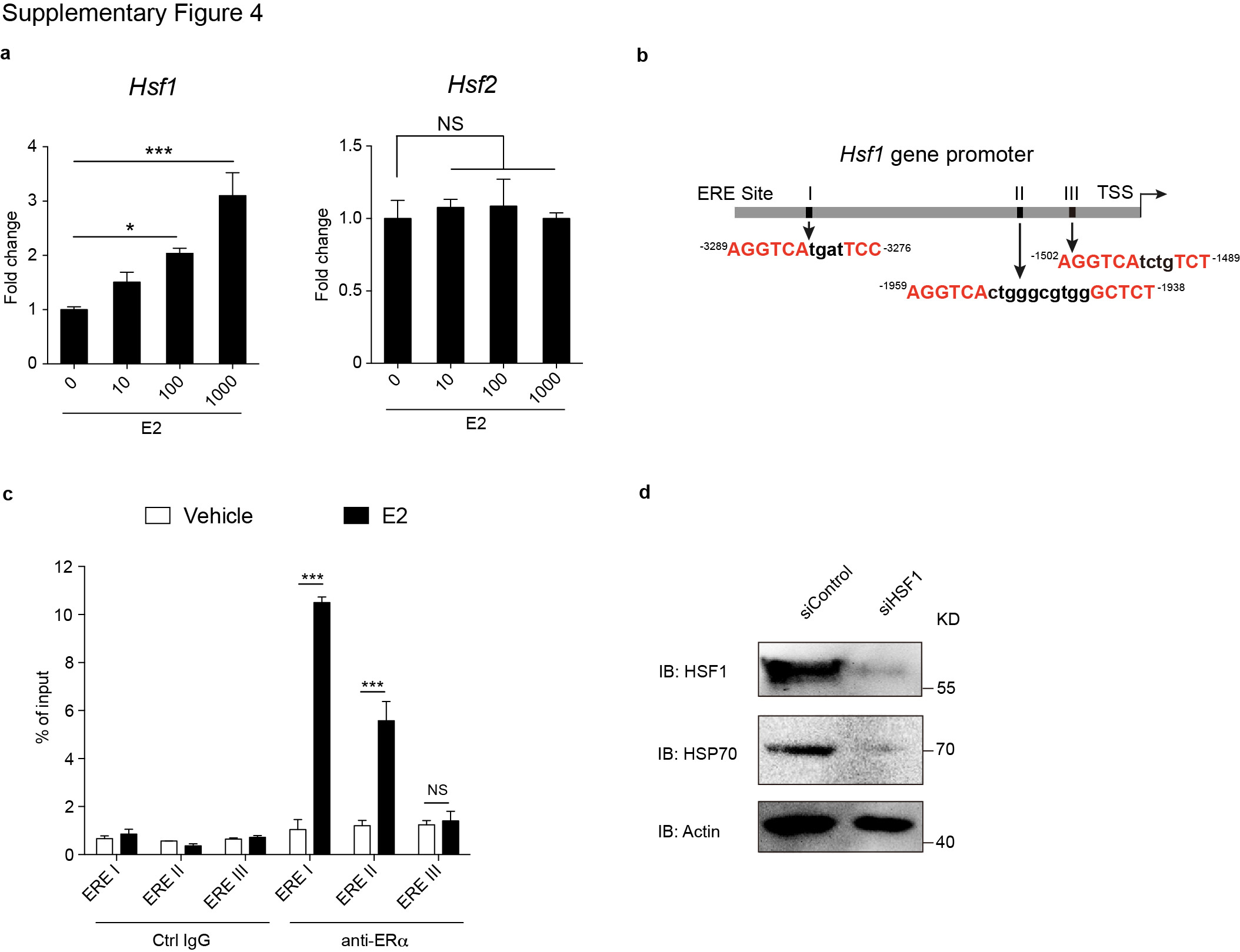


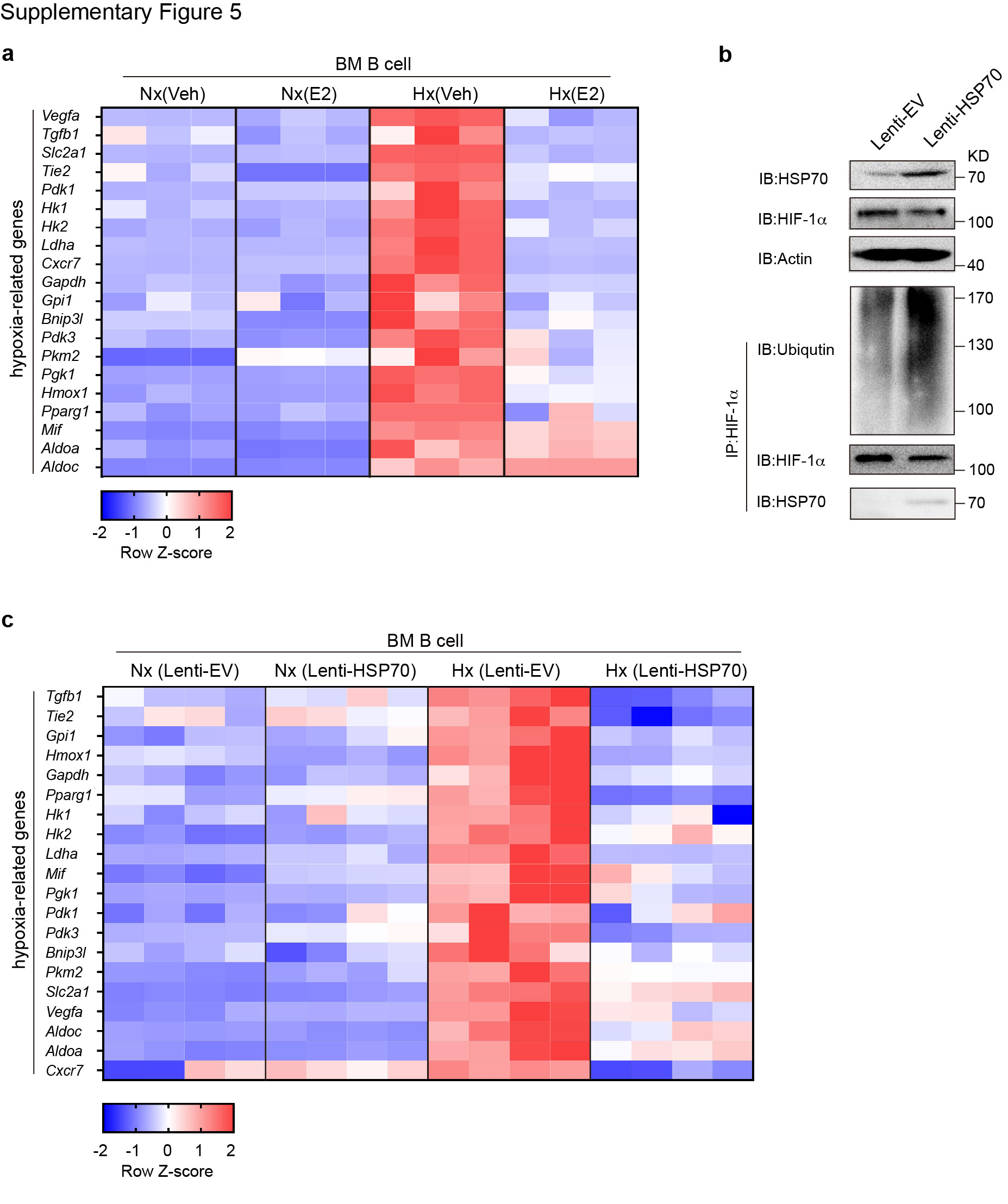


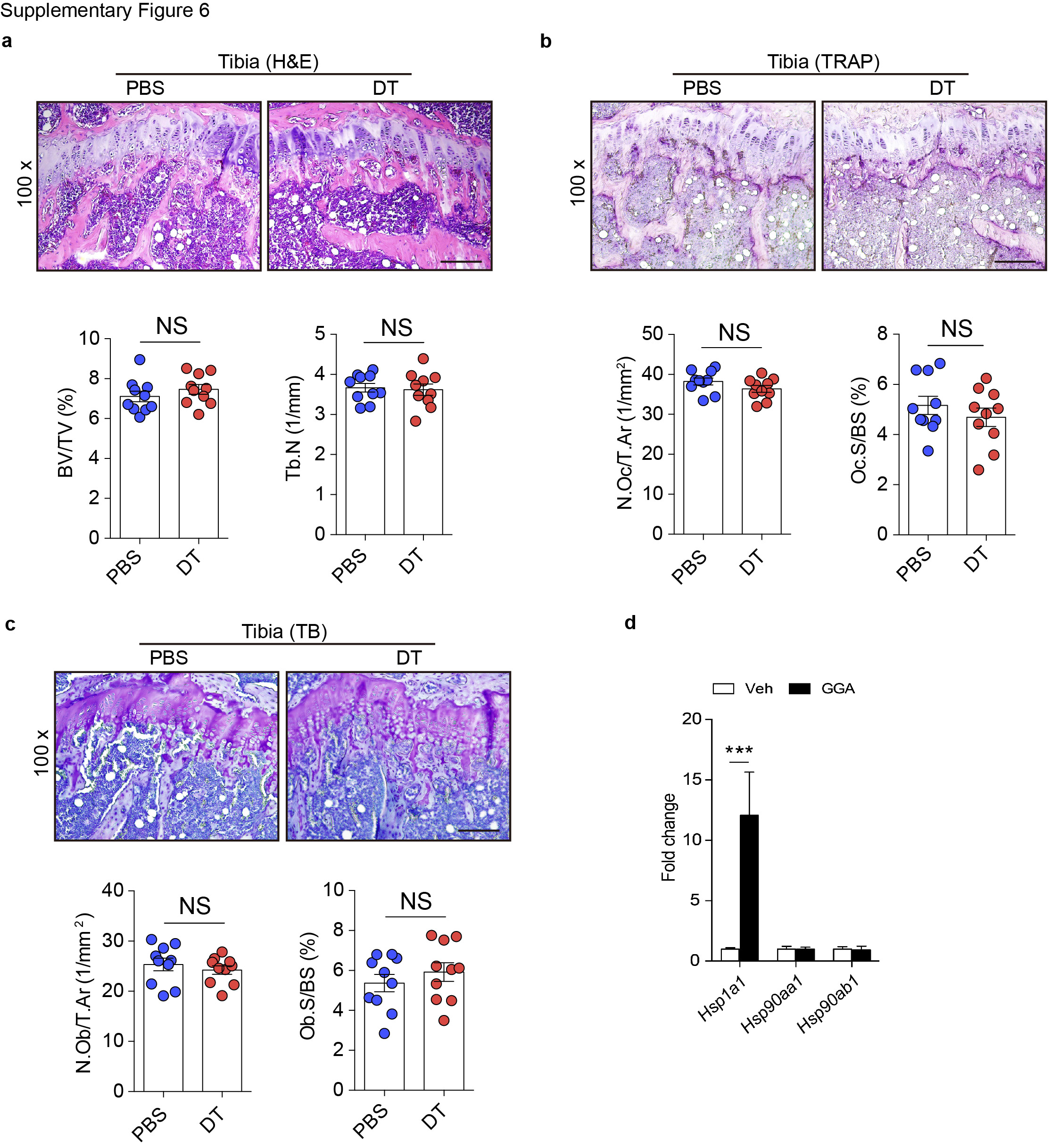


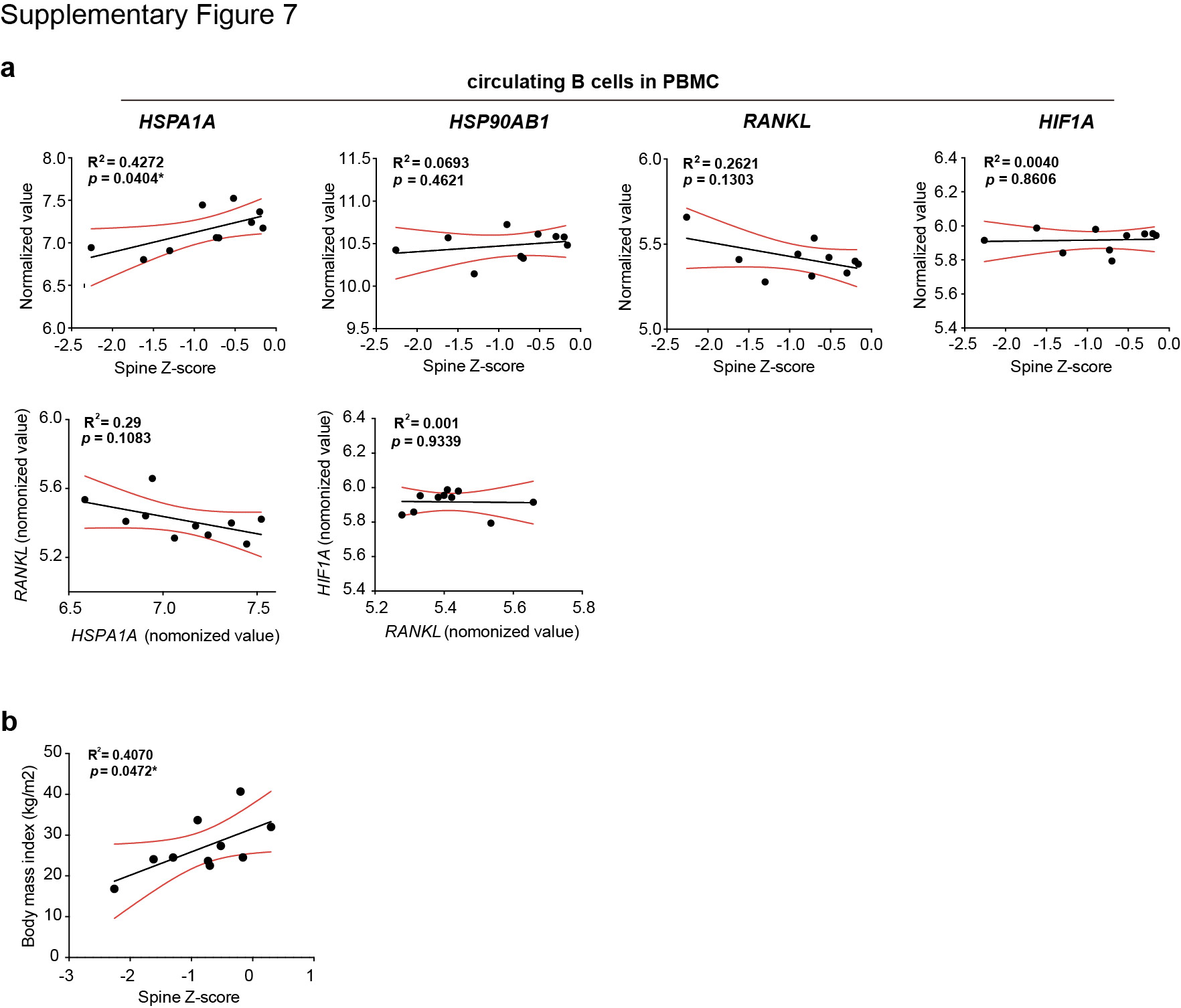


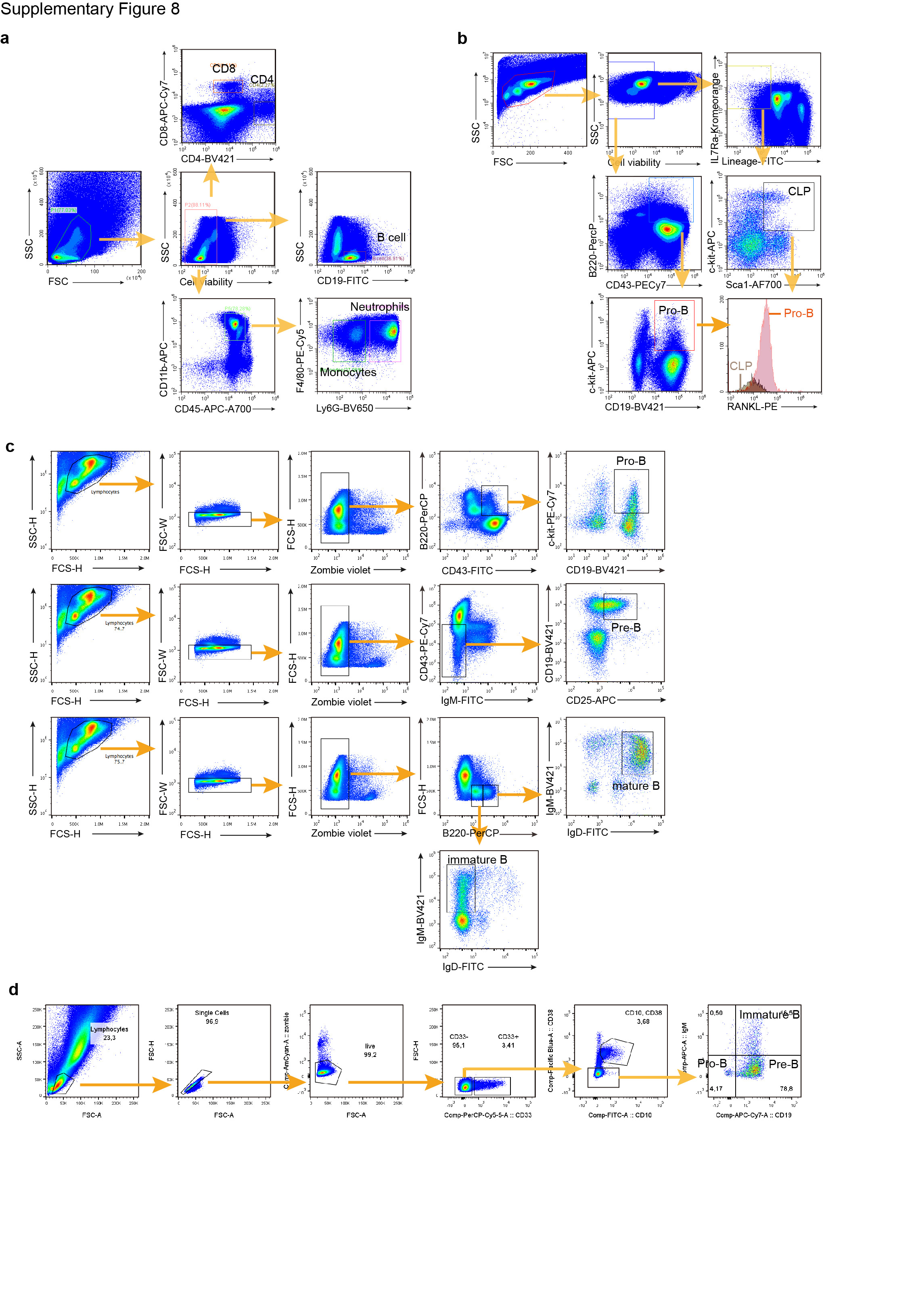


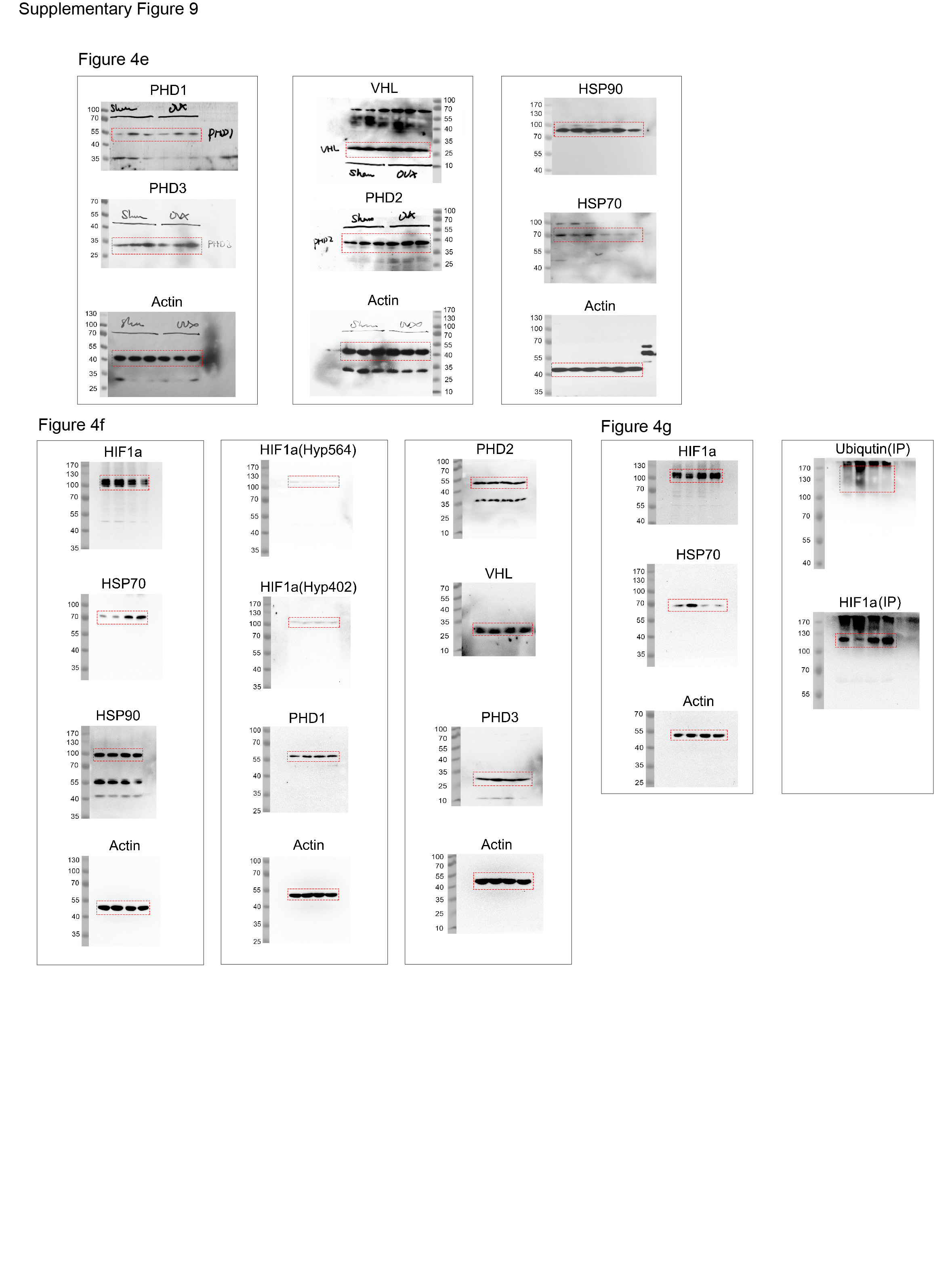


| **Supplementary Table 1** |  |
| --- | --- |
| Gene name | Function |
| *Tnfsf11* | bone resorption, osteoclast differentiation,mammary gland alveolus development |
| *Jup* | cell migration,desmosome assembly,regulation of heart rate by cardiac conduction |
| *Ust* | establishment of cell polarity,regulation of axonogenesis |
| *Gnaz* | G protein-coupled receptor signaling pathway,signal transduction |
| *Kitl* | cell adhesion,embryonic hemopoiesis,ovarian follicle development |
| *Aif1* | actin filament bundle assembly,phagocytosis, engulfment,microglial cell activation |
| *Kalrn* | axonogenesis,modification of postsynaptic actin cytoskeleton,protein phosphorylation |
| *Mylk* | aorta smooth muscle tissue morphogenesis,smooth muscle contraction |
| *Parvb* | actin cytoskeleton reorganization,cell projection assembly,lamellipodium assembly |
| *Cpt1a* | carnitine metabolic process,long-chain fatty acid metabolic process |
| *Fgf1* | angiogenesis,lung development,mesonephric epithelium development |
| *Fgf13* | cerebral cortex cell migration,establishment of neuroblast polarity,microtubule polymerization |
| *Dlg2* | anterograde axonal protein transport,embryo development,neuronal ion channel clustering |
| *Shank2* | brain morphogenesis,dendritic spine morphogenesis,postsynaptic density assembly |
| *Dgki* | diacylglycerol metabolic process,lipid phosphorylation,neurotransmitter secretion |
| *Igf2bp3* | nervous system development,regulation of RNA metabolic process |
| *Mmrn1* | blood coagulation,extracellular matrix structural constituent |
| *Ccdc148* | biological_process,cellular_component |
| *Nr1h3* | apoptotic cell clearance,cholesterol homeostasis,fatty acid biosynthetic process |
| *Fbn1* | glucose metabolic process,heart development,metanephros development |
| *Cnbd2* | spermatogenesis,cAMP binding |
| *Scn4b* | cardiac muscle cell action potential involved in contraction,sodium ion transport |
|  |  |

| **Supplementary Table 2** |  |  |
| --- | --- | --- |
| **QPCR assay** |  |  |
| **Primer pair name** | **Sequence (5'-3')** | **Application** |
| *Hif1a* (For) | CCTGCACTGAATCAAGAGGTTGC | RT-qPCR |
| *Hif1a* (Rev) | CCATCAGAAGGACTTGCTGGCT |  |
| *Trap* (For) | GCGCTAGACTCCGAGAACAT | RT-qPCR |
| *Trap* (Rev) | TGGCCACTTACTACCTGACCCTT |  |
| *Cathk* (For) | AGCAGAACGGAGGCATTGACTC | RT-qPCR |
| *Cathk* (Rev) | CCCTCTGCATTTAGCTGCCTTTG |  |
| *Col1a1* (For) | CCTCAGGGTATTGCTGGACAAC | RT-qPCR |
| *Col1a1* (Rev) | CAGAAGGACCTTGTTTGCCAGG |  |
| *Runx* (For) | CCTGAACTCTGCACCAAGTCCT | RT-qPCR |
| *Runx* (Rev) | TCATCTGGCTCAGATAGGAGGG |  |
| *Rankl* (For) | GTGAAGACACACTACCTGACTCC | RT-qPCR |
| *Rankl* (Rev) | GCCACATCCAACCATGAGCCTT |  |
| *Vhl* (For) | GTTTGTGCCATCCCTCAATGTCG | RT-qPCR |
| *Vhl* (Rev) | ACCTGACGATGTCCAGTCTCCT |  |
| *Hprt* (For) | CTGGTGAAAAGGACCTCTCGAAG | RT-qPCR |
| *Hprt* (Rev) | CCAGTTTCACTAATGACACAAACG |  |
| *Phd1* (For) | ATGGCTCACGTGGACGCAGTAA | RT-qPCR |
| *Phd1* (Rev) | CATTGCCTGGATAACACGCCAC |  |
| *Phd2* (For) | TGAGCAGCATGGACGACCTGAT | RT-qPCR |
| *Phd2* (Rev) | GACATAGCCTGTTCCGTTGCCT |  |
| *Phd3* (For) | CAACTTCCTCCTGTCCCTCATC | RT-qPCR |
| *Phd3* (Rev) | CCTGGATAGCAAGCCACCATTG |  |
| *Hspa1a* (For) | ACAAGTCGGAGAACGTGCAGGA | RT-qPCR |
| *Hspa1a* (Rev) | GTTGTCCGAGTAGGTGGTGAAG |  |
| *Hsp90aa1* (For) | GCTTTCAGAGCTGTTGCGGTAC | RT-qPCR |
| *Hsp90aa1* (Rev) | AAAGGCGGAGTTAGCAACCTGG |  |
| *Hsp90ab1* (For) | CCTGAAGGTCATCCGCAAGAAC | RT-qPCR |
| *Hsp90ab1*(Rev) | GGCGTCGGTTAGTGGAATCTTC |  |
| *Vegfa* (For) | CTGCTGTAACGATGAAGCCCTG | RT-qPCR |
| *Vega* (Rev) | GCTGTAGGAAGCTCATCTCTCC |  |
| *Tgfb1* (For) | TGATACGCCTGAGTGGCTGTCT | RT-qPCR |
| *Tgfb1*  (Rev) | CACAAGAGCAGTGAGCGCTGAA |  |
| *Tie2* (For) | GCTCCAACCAAATGACTTC | RT-qPCR |
| *Tie2* (Rev) | CACGAGGTTGGTTTACTGA |  |
| *Hk2* (For) | GGAGAGCACGTGTGACGAC | RT-qPCR |
| *Hk2* (Rev) | GATGCGACAGGCCACAGCA |  |
| *Ldha* (For) | CACAAGCAGGTGGTGGACAG | RT-qPCR |
| *Ldha* (Rev) | AACTGCAGCTCCTTCTGGATTC |  |
| *Cxcr7* (For) | GACCGCTATCTCTCCATCACCT | RT-qPCR |
| *Cxcr7* (Rev) | GTTGGAAGCAGATGTGACCGTC |  |
| *Gpi1* (For) | GTTGCCTGAAGAGGCCAGG | RT-qPCR |
| *Gpi1* (Rev) | GCTGTTGCTTGATGAAGCTGATC |  |
| *Bnip3l* (For) | GCATGAGGAAGAGTGGAGCCAT | RT-qPCR |
| *Bnip3l* (Rev) | AAGGTGTGCTCAGTCGTTTTCCA |  |
| *Pdk3* (For) | CCGTCGCCACTGTCTATCAAAC | RT-qPCR |
| *Pdk3* (Rev) | CTCTCATGGTGTTAGCCAGTCG |  |
| *Pkm2* (For) | CAGGAGTGCTCACCAAGTGG | RT-qPCR |
| *Pkm2* (Rev) | CATCAAGGTACAGGCACTACAC |  |
| *Pgk1* (For) | TTGGACAAGCTGGACGTGAA | RT-qPCR |
| *Pgk1* (Rev) | GCAGCCTTGATCCTTTGGTTG |  |
| *Pparg1* (For) | AGAGGTCCACAGAGCTGATTC | RT-qPCR |
| *Pparg1* (Rev) | TTTAAAAACAAGACTACCCTTTACTGAAATT |  |
| *Mif* (For) | GAACCGCAACTACAGTAAGCTGC | RT-qPCR |
| *Mif* (Rev) | ACGTTGGCAGCGTTCATGTCGT |  |
| *Aldoc* (For) | GGCAGAGATGAACGGGCTTG | RT-qPCR |
| *Aldoc* (Rev) | GGCGATGTAGAGGGACTGTG |  |
| *Gapdh*(For) | CATCACTGCCACCCAGAAGACTG | RT-qPCR |
| *Gapdh*(Rev) | ATGCCAGTGAGCTTCCCGTTCAG |  |
| *Hmox1*(For) | CACTCTGGAGATGACACCTGAG | RT-qPCR |
| *Hmox1*(Rev) | GTGTTCCTCTGTCAGCATCACC |  |
| *Epo* (For) | GACAAAGCCATCAGTGGTCTACG | RT-qPCR |
| *Epo* (Rev) | GCAGAAAGTATCCACTGTGAGTG |  |
| *Hk1*(For) | GAAAGGAGACCAACAGCAGAGC | RT-qPCR |
| *Hk1* (Rev) | TTCGTTCCTCCGAGATCCAAGG |  |
| *Aldoa* (For) | CACGAGACACTGTACCAGAAGG | RT-qPCR |
| *Aldoa*(Rev) | TTGTCTCGCCATTGGTTCCTGC |  |
| *Hsf1* (For) | GCACACTCTGTGCCCAAGTATG | RT-qPCR |
| *Hsf1* (Rev) | AGCTGGTGACAGCATCAGAGGA |  |
| *Hsf2* (For) | CCAACGAGTTCATCACCTGGAG | RT-qPCR |
| *Hsf2* (Rev) | GTTGTCTCACAAAGCTCGCCATG |  |
| *HIF1A* (For) | TATGAGCCAGAAGAACTTTTAGGC | RT-qPCR |
| *HIF1A* (Rev) | CACCTCTTTTGGCAAGCATCCTG |  |
| *RANKL* (For) | GCCTTTCAAGGAGCTGTGCAAAA | RT-qPCR |
| *RANKL*  (Rev) | GAGCAAAAGGCTGAGCTTCAAGC |  |
| *ACTIN* (For) | CACCATTGGCAATGAGCGGTTC | RT-qPCR |
| *ACTIN* (Rev) | AGGTCTTTGCGGATGTCCACGT |  |
|  |  |  |
|  |  |  |
| **ChIP assay** |  |  |
| **Primer pair name** | **Sequence (5'-3')** |  |
| HRE I (For) | CCGTCTTATTGGCCCTCAGC |  |
| HRE I (Rev) | GGGGATGATGGAACAGTCAGG |  |
| HRE II (For) | ACATGCCGGAGGAAAAGGATG |  |
| HRE II (Rev) | GCCTTATGCTGAGGGCCAATA |  |
| ERE I (For) | CGATCTAGGCCTGGCTTCTTG |  |
| ERE I (Rev) | TGGTTTACACTTCAGTGGCCC |  |
| ERE II (For) | CGATCTAGGCCTGGCTTCTTG |  |
| ERE II (Rev) | TGGTTTACACTTCAGTGGCCC |  |
| ERE III (For) | CAGCCCTAGAGGCCACTTTAC |  |
| ERE III (Rev) | CAGTCACATGCTTCCTCCAGT |  |
|  |  |  |
|  |  |  |
| ***Rankl* Promoter regions cloning** | |  |
| **Primer pair name** | **Sequence (5'-3')** |  |
| HIF1α binding region I | CCGAGCTCACGTGTTGCTTTGGGTGTTG |  |
|  | CCCTCGAGGACAGCCTGGTCCCATCTTT |  |
| HIF1α binding region II | CCGAGCTCGCGCGTGTGCGTGCT |  |
|  | CCCTCGAGCTTGAGGACCTGAATTTGACCAG |  |
